# *Escherichia coli* CspA stimulates translation in the cold of its own mRNA by promoting ribosome progression

**DOI:** 10.3389/fmicb.2023.1118329

**Published:** 2023-02-09

**Authors:** Anna Maria Giuliodori, Riccardo Belardinelli, Melodie Duval, Raffaella Garofalo, Emma Schenckbecher, Vasili Hauryliuk, Eric Ennifar, Stefano Marzi

**Affiliations:** ^1^School of Biosciences and Veterinary Medicine, University of Camerino, Camerino, Italy; ^2^Architecture et Réactivité de l’ARN, CNRS 9002, Université de Strasbourg, Strasbourg, France; ^3^Department of Experimental Medical Science, Lund University, Lund, Sweden; ^4^Institute of Technology, University of Tartu, Tartu, Estonia

**Keywords:** cold-shock, translation regulation, CspA, RNA chaperone, ribosome

## Abstract

*Escherichia coli* CspA is an RNA binding protein that accumulates during cold-shock and stimulates translation of several mRNAs—including its own. Translation in the cold of *cspA* mRNA involves a cis-acting thermosensor element, which enhances ribosome binding, and the trans-acting action of CspA. Using reconstituted translation systems and probing experiments we show that, at low temperature, CspA specifically promotes the translation of the *cspA* mRNA folded in the conformation less accessible to the ribosome, which is formed at 37°C but is retained upon cold shock. CspA interacts with its mRNA without inducing large structural rearrangements, but allowing the progression of the ribosomes during the transition from translation initiation to translation elongation. A similar structure-dependent mechanism may be responsible for the CspA-dependent translation stimulation observed with other probed mRNAs, for which the transition to the elongation phase is progressively facilitated during cold acclimation with the accumulation of CspA.

## Introduction

Cold is a physical stress that may influence conformation, flexibility, topology, and interactions of every macromolecule in the cell. When subjected to abrupt temperature downshifts, mesophilic bacteria stop growing for several minutes until acclimation is established and growth resumes at lower temperature (for a review, see Gualerzi et al., 2003; Weber and Marahiel, 2003, Barria et al., 2013; Giuliodori, 2016). This cold acclimation phase is accompanied by drastic reprogramming of gene expression: whereas RNA, protein, and lipid synthesis rates are in general reduced, the production of a small set of cold-shock (CS) proteins transiently increases (Gualerzi et al., 2003; Phadtare, 2004; Giuliodori, 2016). These CS proteins are mainly transcription and translation factors as well as proteins involved in RNA structure remodeling, such as RNA chaperones, RNA helicases, and exoribonucleases (Jones et al., 1992; Yamanaka et al., 1998; Bae et al., 1999; Gualerzi et al., 2003). The timing of their induction suggests that the expression of some CS genes might depend on the synthesis of early CS proteins (Weber and Marahiel, 2003; Zhang et al., 2018), which are regulated at the transcriptional and more so, at post-transcriptional levels (Goldenberg et al., 1997; Gualerzi et al., 2003). In fact, low temperature impairs translation, affecting both the initiation (Broeze et al., 1978; Farewell and Neidhardt, 1998; Zhang et al., 2018) and the elongation (Friedman and Weinstein, 1964; Farewell and Neidhardt, 1998; Zhang et al., 2018) phases. The ability of the translational machinery to synthesize proteins under these unfavorable conditions relies on cis-acting elements encoded in mRNAs, whose existence was demonstrated *in vivo* (Mitta et al., 1997; Yamanaka et al., 1999; Etchegaray and Inouye, 1999; Zhang et al., 2018) and using *in vitro* assays (Giuliodori et al., 2010, 2019). Trans-acting factors also play an important role in this process, such as the initiation factors IF1 and IF3, and the protein CspA, whose levels specifically increase during cold-shock (Giuliodori et al., 2004, 2007; Di Pietro et al., 2013; Zhang et al., 2018).

CspA, a member of the CS protein (Csp) family, is the most well-studied *Escherichia coli* CS protein (Goldstein et al., 1990; Yamanaka et al., 1998). Out of the nine paralogues of this family, seven are cold-inducible (CspA, CspB, CspE, CspF, CspG, CspH and CspI) and two are expressed only at 37°C (CspC and CspD; Zhang et al., 2018; Giuliodori et al., 2019). Furthermore, expression of CspF and CspH is also induced upon urea challenge (Withman et al., 2013). To generate a cold-sensitive phenotype, four out of the nine *csp* genes must be knocked out in *E. coli* genome, and this cold-sensitive phenotype can be reverted by overexpressing any of the *csp* members, with the exception of *cspD* (Xia et al., 2001). CspA is the most abundant protein expressed during cold acclimation, accounting for up to 10% of the total proteins (Brandi et al., 1999), with intracellular CspA concentration reaching 100 μM (Bae et al., 1999; Brandi et al., 1999). It is a small protein of 70 amino acids comprised of a single OB fold domain, similar to S1 domain (Newkirk et al., 1994; Schindelin, 1994). The protein preferentially binds single strand regions of RNA and DNA (Jiang et al., 1997), with low binding selectivity and affinity (dissociation constants in the μM range; Jiang et al., 1997; Phadtare and Inouye, 1999; Lopez and Makhatadze, 2000), typical of RNA chaperone proteins, which bind RNAs only transiently (Mayer et al., 2007; Rajkowitsch and Schroeder, 2007; Duval et al., 2017). Therefore, it is expected that in cold-stressed cells several molecules of CspA could bind simultaneously to an individual target mRNA (Ermolenko and Makhatadze, 2002; Zhang et al., 2018) modulating its structures induced/stabilized by low temperatures. Indeed, CspA has been proposed to regulate translation in the cold acting as RNA chaperone (Jiang et al., 1997; Giuliodori et al., 2004; Zhu et al., 2008; Rennella et al., 2017; Zhang et al., 2018). Interestingly, also *cspA* mRNA encoding CspA was suggested to be a *bona fide* target of CspA-mediated regulation (Jiang et al., 1997).

In *E. coli* the extent of cold-shock induction of *cspA* mRNA is growth phase-dependent (Brandi et al., 1999). When cells are subjected to cold-shock during mid-late exponential growth, they abundantly transcribe and translate *cspA* mRNA *de novo* to increase the level of the protein. In this condition, transcription at low temperature allows *cspA* mRNA to adopt an active structure ready for translation (Giuliodori et al., 2010). In this structure, the Shine-Dalgarno sequence (SD) is exposed into a large loop and the whole ribosome binding site is embedded into two unstable RNA helices containing bulges and made up mainly by weak A-U or G·U pairs. Conversely, when cells are subjected to cold-shock in the early stage of growth, they predominantly use CspA and its transcript synthetized at 37°C, at least at the beginning of the cold adaptation phase. In this case, *cspA* mRNA adopts a close structure which is less translationally active having both SD and AUG hindered into stable RNA helices (Giuliodori et al., 2010).

In the present work, we have characterized the mechanism of translational activation of *cspA* mRNA by CspA using a cell-free reconstituted translation system and probing methods. Using the two *cspA* mRNA forms in translation assays performed at low temperature, we demonstrate that CspA binds to the two *cspA* mRNA conformations at various positions but specifically promotes translation of the mRNA with the more closed structure. CspA binding *per se* does not promote unwinding of the stably folded regions of *cspA* mRNA, but assists the progression of elongating ribosomes and their transition from translation initiation to translation elongation. Similarly, we demonstrate by cross-linking experiments that CspA binds to other mRNAs, preferentially in a position located downstream from the initiation codon, thus stimulating the translation of some of these transcripts. Indeed, analysis of available ribosome profiling data during cold acclimation shows that these CspA-dependent mRNAs have ribosomes stalled on the initiation codons, which progress to translation elongation with the cellular accumulation of CspA. Eventually, we proposed a model that takes into account these results and explain the translation activity of CspA upon cold shock.

## Materials and methods

### General preparations and buffers

*Escherichia coli* MRE600 70S ribosomes, S100 post-ribosomal supernatant, 30S ribosomal subunits and purified initiation factors IF1, IF2, and IF3 were prepared as described previously (Giuliodori et al., 2004 and 2007).

The following buffers were used:

*Buffer A*: 25 mM tris–HCl, pH 8.5, 5% glycerol, 100 mM NaCl, 0.025% Nonidet P40; *Buffer B*: 25 mM tris–HCl, pH 8, 1.3 M NaCl, 5% glycerol, 6 mM β-mercaptoethanol, 0.1 mM PMSF, 0.1 mM benzamidine; *Buffer C*: 25 mM tris–HCl, pH 8.0, 700 mM NaCl, 5% glycerol, 6 mM β-mercapto-ethanol, 0.1 mM PMSF, 0.1 mM benzamidine; *Buffer D*: 25 mM tris–HCl, pH 8.0, 300 mM of NaCl, 5% glycerol, 20 mM Imidazole, 6 mM β-mercaptoethanol, 0.1 mM PMSF, 0.1 mM benzamidine; *Buffer E*: 25 mM tris–HCl, pH 8.0, 300 mM of NaCl, 5% glycerol, 300 mM Imidazole, 6 mM β-mercaptoethanol, 0.1 mM PMSF, 0.1 mM benzamidine; *Buffer F*: 25 mM tris–HCl, pH 8.0, 100 mM NaCl, 5%glycerol, 6 mM β-mercaptoethanol, 0.1 mM PMSF, 0.1 mM benzamidine; *Buffer G*: 25 mM tris–HCl, pH 8.0, 300 mM NaCl, 5% glycerol, 6 mM β-mercaptoethanol, 0.1 mM PMSF, 0.1 mM benzamidine; *Buffer H*: 20 mM tris–HCl, pH 7.1, 10 mM NH_4_Cl, 1 mM MgCl_2_, 10% glycerol, 0.1 mM EDTA, 6 mM β-mercaptoethanol; *Buffer I*: 20 mM Hepes-KOH, pH 7.5, 10 mM MgCl_2_, 50 mM KCl; *Buffer L*: 10 mM Tris–HCl pH 7.5, 60 mM NH_4_Cl, 1 mM DTT, 7 mM MgCl_2_; *Buffer M*: 20 mM Tris–HCl pH 7.5, 60 mM KCl, 1 mM DTT, 10 mM MgCl_2_; *Buffer N*: 20 mM Na-cacodylate, pH 7.2, 10 mM MgCl_2_, 50 mM KCl; *Buffer O*: 20 mM Tris–HCl pH 7.5, 60 mM KCl, 40 mM NH_4_Cl, 3 mM DTT, 10 mM MgCl_2_, 0.002 mg/ml BSA; ITC buffer: 20 mM Tris–HCl pH 7.1, 10 mM NH_4_Cl, 7 mM MgCl_2_, 10% glycerol, 0.1 mM EDTA, 6 mM β-mercaptoethanol.

### Molecular cloning, expression, and purification of CspA

The coding region of *E. coli cspA* gene was amplified by PCR from the pUT7cspA construct (Giuliodori et al., 2010) using the forward primer G655 5′-CATGCCATGGCCGGTAAAATGACTGGTATCG-3′ and the reverse primer G656 5′-CGGGATCCTTACAGGCTGGTTACGTTAC-3′ and cloned into the pETM11 vector (Dümmler et al., 2005) using NcoI and BamHI restriction sites. Because the introduction of the NcoI restriction site had changed the second amino acid of the CspA sequence, after the molecular cloning the wt sequence was restored by mutagenesis using the QuikChange Site-Directed Mutagenesis Kit (Agilent Technologies, Inc., Santa Clara, CA), the pETM11-CspA plasmid as DNA template and the mutagenic primers G670 5′-TTTCAGGGCGCCATGTCCGGTAAAATGACTG-3′ and G671 5′-CAGTCATTTTACCGGACATGGCGCCCTGAAA-3′.

Overproduction of protein CspA was induced into a culture of *E. coli* BL21 (DE3)/pLysS cells grown in LB medium at 37°C till OD_600_ = 0.4 by the addition of 1 mM of isopropylbeta-D-1-thiogalactopyranoside (IPTG). After transferring the culture to 20°C for 12 h, cells were harvested by centrifugation and the pellet resuspended in Buffer A and stored at −80°C. After thawing, cells were diluted in an equal volume of Buffer B and lysed by sonication. The resulting cell extract, cleared by centrifugation, was loaded onto a nickel-nitrilotriacetic acid (Ni-NTA) chromatographic column equilibrated in Buffer C. After washing in Buffer D, protein CspA was eluted using Buffer E, pooled and dialyzed against Buffer F. To remove the His-Tag sequence, 15 mg of CspA were incubated for 4 h at 20°C with the His-Tag TEV protease (Kapust et al., 2002). At the end of the incubation, the concentration of NaCl was increased to 300 mM and the cleaved CspA was loaded onto a Ni-NTA column equilibrated in Buffer G. The flow-through, containing CspA with no His-Tag, was dialysed overnight at 4°C against Buffer H. Then, CspA was concentrated by centrifugation in Microcon tubes (Amicon-Millipore) with 3 KDa cut-off at 13.8 krcf, 4°C, until the concentration was ≥400 μM and stored at −80°C in small aliquots. The purity of CspA protein was checked by 18% SDS-PAGE.

### mRNA preparation

The DNA templates used *for in vitro* transcription of the various mRNAs were constructed as specified (Giuliodori et al., 2010; Di Pietro et al., 2013). All mRNAs obtained by *in vitro* transcription with T7 RNA polymerase were purified and labelled as described (Giuliodori et al., 2010).

### Translation assays

Before use, the mRNAs were denatured at 90°C for 1 min in RNase free H_2_O and renatured for 15 min at 15°C or 37°C in Buffer I. When required, CspA was added after renaturation at the concentrations indicated in the figures.

*In vitro* translation reactions were carried out in 30 μL containing 20 mM Tris–HCl, pH 7.7, 12 mM Mg acetate, 80 mM NH_4_Cl, 2 mM DTT, 2 mM ATP, 0.4 mM GTP, 10 mM phosphoenolpyruvate, 0.025 μg of pyruvate kinase/μL reaction, 200 μM of each amino acid (minus Alanine), 5 μM [^3^H] Alanine (309 mCi/mmol), 50 mM cold Alanine, 0.12 mM citrovorum (Serva) and 0.4 U/μL of RNasin (Promega). The reaction mixture also contained 30 pmoles of *in vitro* transcribed mRNAs and either the amount of S30 crude extracts corresponding to 20 pmoles of 70S ribosomes or 30 pmoles of purified 70S ribosomes, 15 pmoles of purified Initiation Factors IF1, IF2 and IF3, and 2 μL of S100 post-ribosomal supernatant. After incubation for the indicated times and temperatures, samples (15 μL) were withdrawn from each reaction mixture and the incorporated radioactivity determined by hot-trichloroacetic acid (TCA) method. The pmoles of protein synthesized were calculated based on the specific activity of [^3^H] Alanine and the number of Ala present in the analyzed proteins.

Initiation complex (IC) formation assays (filter binding) were carried out in 30 μL of Buffer L using 0.5 μM 30S ribosomal subunits either alone (for the 30S IC) or in the presence of 1 μM of 50S subunits (for the 70S IC), 0.5 μM ^35^S-fMet-tRNA, 0.5 mM GTP, 0.5 μM IF1, 0.5 μM IF2, 0.5 μM IF3, 1 μM *cspA* and 0.4 U/ μL of RNasin (Promega). Binding of ^32^P-labelled *cspA* mRNA to 30S subunits was performed in 40 μL of Buffer L containing 20 pmoles of *cspA* mRNA and 9,000 cpm of [^32^P] *cspA* mRNA, 0.4 U/μL of RNasin (Promega), 20 pmoles of 30S subunits and either 30 pmoles of tRNA_i_^fMet^ or 30 pmoles of fMet-tRNA_i_^fMet^ and 20 pmoles of IF1, IF2 and IF3. After 30 min incubation at 15°C, the amount of initiation complex formed was determined either by filtration through 96-multiscreen-HTS-HA Millipore plates (mRNA binding) or by nitrocellulose filtration (30S and 70S IC), followed by liquid scintillation counting.

The toeprinting assay was performed essentially as described (Fechter et al., 2009). The reaction was carried out in 10 μL of Buffer M containing 0.4 U/μL of RNasin (Promega) in the presence of 0.02 μM *cspA* mRNA, 4 μM tRNA_i_^fMet^, 50 μM each of dNTPs, ^32^P-labeled oligo csp2 (5′-CGAACACATCTTTAGAGCCAT-3′), and 0.2 μM of *E. coli* 30S subunits. The reaction mixtures were incubated for 30 min at 15°C. Primer extension was conducted with 4 units of Avian Myeloblastosis Virus (AMV) reverse transcriptase (Sigma) for 1 h at 15°C. The reaction products were analyzed on 8% PAGE-urea gel.

### RNA footprinting assays

Before use, the RNAs were denatured at 90°C for 1 min in RNase-free H_2_O and renatured for 15 min at 15°C or 37°C in the buffers used for enzymatic probing or hydroxyl radical cleavage experiments.

Enzymatic probing was carried out on ^32^P-end-labeled transcripts (50,000 cpm) essentially as described earlier (Giuliodori et al., 2010) after incubating the renatured mRNA with the amounts of CspA indicated in the figure legends.

Probing by hydroxyl radical cleavage was performed essentially as described (Fabbretti et al., 2007). CspA was allowed to bind *cspA* mRNA in 40 μL of Buffer N by incubating 10 pmoles of renatured mRNA with the indicated amounts of protein CspA in the presence of 0.4 U/μL of RNasin (Promega). After 15 min at 15°C, H_2_O_2_ was added (0.15% final concentration) and the cleavage started by adding Fe(II)-EDTA (3 mM final concentration). Cleavage was allowed to proceed for 15 s at 15°C before addition of 260 μL quenching solution containing 0.3 M Na acetate (pH 5.2) in absolute ethanol. The precipitated samples were resuspended in H_2_O, extracted with phenol-chloroform and re-precipitated with cold 0.3 M Na acetate (pH 5.2) in absolute ethanol. These reaction products, resuspended in 3 μL of sterile H_2_O, were then subjected to primer extension analysis as described earlier (Fabbretti et al., 2016) using cspA1, csp2 and cspA3 primers (Giuliodori et al., 2010).

### CspA-RNA cross-linking

For the CspA-RNA cross-linking experiments, 0.02 μM of the ^32^P-labeled primers indicated in the figure legends were mixed with 0.35 μM of the corresponding mRNAs. After a denaturation step at 90°C for 1 min, the samples were incubated at either 15°C or 37°C for 10 min in Buffer I containing 0.4 U/μL of RNasin (Promega). Following renaturation, the reaction mixtures were dispensed in tubes containing increasing amounts of purified CspA (reaction volumes: 10 μL) and the protein was allowed to bind for 10 min at the indicated temperatures. Subsequently, the samples were transferred to an ice-cold plate and U.V. irradiated for 2 min using the GS Gene-linker BioRad (180 mJ, 254 nm bulbs at 12 cm from the U.V. source). The cross-linked RNA was primer-extended using AMV Reverse Transcriptase as previously described (Giuliodori et al., 2010).

### Isothermal titration calorimetry

All samples were dialyzed against ITC buffer using centrifugal filter units (Centricon, Merck Millipore), 3 K for CspA and 100 K for *E. coli* ribosome. ITC experiments were done on a MicroCal PEAQ-ITC microcalorimeter (Microcal-Malvern Panalytical, Malvern, UK). Experiments were done by successive injections of CspA in 30S, 50S or 70S solution at three different temperatures (15°C, 25°C, and 35°C). Titration of *E. coli* ribosomes with CspA was performed by sequential injections of 2 or 1 μL CspA in the cell containing 200 μL of 70S ribosomes, 30S subunits or 50S subunits, present at the concentrations indicated in the panels of Supplementary Figures S2, S3. Control experiment was performed using initiation factor IF1 and 30S subunits. The CspA /ribosome or IF1/ribosome molar ratios attained in the titration experiments are shown in the panels of Supplementary Figures S2, S3. Data were analyzed with MicroCal PEAQ-ITC Analysis Software.

### RelE walking assays

RelE toxin was expressed and purified as described earlier (Andreev et al., 2008). Ribosome progression on *cspA* mRNA was monitored by analysing the amount of RelE cleavages obtained by ribosomes paused at different sites on the mRNA. *In vitro* translation was carried out in 10 μL using the PURExpress kit (NEB) according to the commercial protocol in the presence of a mix of ^32^P-radiolabeled *cspA* mRNA (200,000 cpm/μL) and cold *cspA* mRNA (0.8 μM), previously folded at 15°C or 37°C. When present, CspA was added at concentration of 30 μM. The reaction was incubated 2 h at 15°C, and blocked by addition of chloramphenicol (1 mM) and different concentration of RelE as indicated in the corresponding figure, for 15 min at 15°C. The RNA fragments were then phenol extracted, subjected to 8% PAGE-urea and revealed by autoradiography. Quantification of each band was done using ImageQuant TL (GE Healthcare) and signal normalization was done using the sum of the quantization of all the bands present in each lane.

### RNA electrophoretic mobility shift assay

Radiolabelled purified *187cspA* RNA (Giuliodori et al., 2010), 50,000 cps/sample, at concentration < 1 pM, was denatured and renaturated at 15°C or 37°C, as described above. For each experiment, increasing concentrations of purified CspA (30–211 μM) were added to the 5′ end labelled *187cspA* RNA in a total volume of 10 μL in Buffer O containing 0.4 U/μL of RNasin (Promega). Complex formation was performed at 15°C or 37°C for 15 min. After incubation, 10 μL of glycerol blue (40% glycerol, 0.05% Xylene Cyanol, 0.05% Bromophenol Blue) was added and the samples were loaded on a 10% PAGE under non-denaturing conditions (1 h, 300 V, 4°C).

### Steady-state fluorescence spectroscopy

To measure binding affinity between CspA and different RNA oligonucleotides, intrinsic tryptophan fluorescence quenching experiments with 1 μM of CspA and increasing amount of RNA oligonucleotides was performed in Buffer H. Fluorescence measurements were performed in quartz cells at 20 ± 0.5°C on a Fluoromax-4 fluorimeter (HORIBA Jobin-Yvon Inc., NJ., USA). The excitation wavelength was set at 295 nm for selective excitation of tryptophan residues and the emission wavelength was scanned from 305 to 450 nm. The peak of emission at 351 nm was used to measure the quenching effect. Increasing amounts of RNA (from 0.05 μM to 18 μM as shown in Supplementary Figure S10) were added and the quartz cell was rapidly homogenized before fluorescence emission measurements. Fluorescence intensities were corrected for buffer fluorescence and dilution effects. Binding parameters were calculated as described (Dubois et al., 2018).

### 3D model of CspA-target RNA interaction

CspA crystal structure (pdb file 1MJC; Schindelin et al., 1993) was superposed to the crystal structure of *B. subtilis* CspB in complex with an eptanucleotide RNA oligo (GUCUUUA; pdb file 3PF4 Sachs et al., 2012). Oligo 1 (AACUGGUA) sequence was then modeled on the RNA structure by Assemble2 software (Jossinet et al., 2010).

### Analysis of ribosome profiling data

Ribosome profiling data at 15 min, 30 min, and 2 h after temperature down-shift (10°C), were obtained from GEO series GSE103421 (Zhang et al., 2018). Raw data have been trimmed of adapter sequences and bad quality reads, before being aligned on *E. coli* genome (NC_000913.3) and visualized with the IGV genome browser (Thorvaldsdóttir et al., 2013). To compare ribosome densities at initiation and stop codons (30 nts window) of specific coding sequences (CDSs) in the three experiments (15 min, 30 min, and 120 min after cold-shock), the read densities have been normalized on the average ribosome density of the whole CDSs.

## Results

### *CspA* stimulates the translation of the *cspA* mRNA closed conformer during cold shock

**Figure 1:**
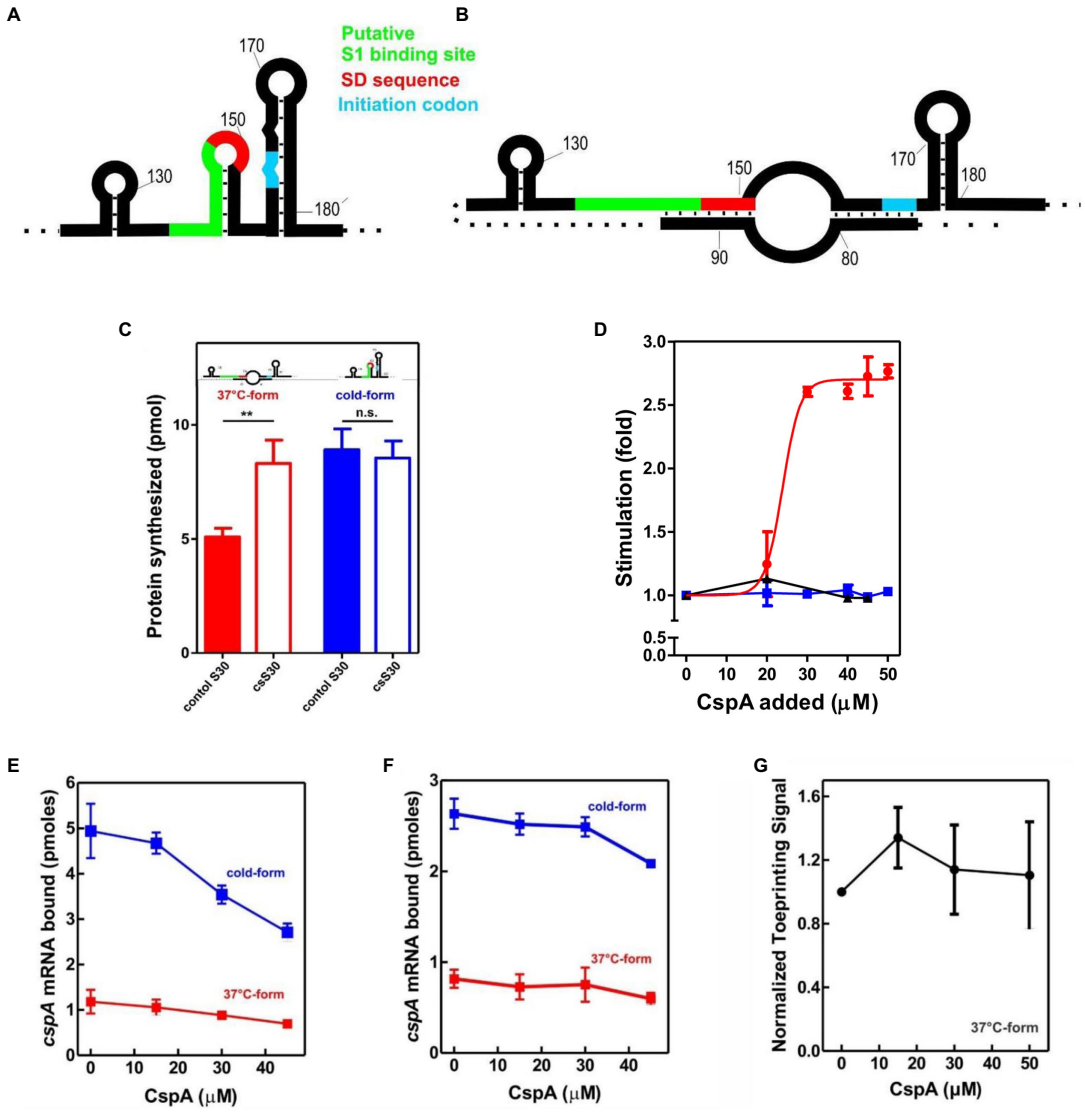
Effect of CspA on *cspA* mRNA translation at low temperature. Schematic representation of the secondary structures of the Translation Initiation Region (TIR) of the **(A)** cold-form and **(B)** 37°C-form of *cspA* mRNA. The SD sequence, the start codon and the putative S1-binding site are indicated in red, light blue, and green, respectively (Giuliodori et al., 2010). **(C)** *In vitro* translation with control (solid bar) and cold-shock (open bar) S30 extracts; the experiment was carried out with *cspA* mRNA folded in the cold-form (blue bar) or in the 37°C-form (red bar). Samples were taken 15 min after incubation at 15°C. The data are the average of four experimental points and the error bars represent the standard error. **(D)** *In vitro* translation with control 70S and S100 in the presence of the indicated amounts of purified CspA at 15°C with the cold-form (blue) or the 37°C-form (red) of *cspA* mRNA. The reaction was also performed at 37°C only with the 37°C-form (black line). The data points are the average of two independent experiments and the error bars represent the standard error. The effect of increasing amounts of CspA on the binding of *cspA* mRNA on the 30S subunits was monitored at 15°C by filter binding of ^32^P-labelled *cspA* mRNAs folded in the cold-form (blue) or in the 37°C-form (red) in the absence **(E)** or in the presence **(F)** of IFs and fMet-tRNA_i_^Met^. The effect of increasing amount of CspA on the initiation phase of translation at 15°C was also investigated by analyzing **(G)** the localization of 30S on the translation start site of *cspA* mRNA by toeprint assay. The data points in panels **(E)** and **(F)** are the average of triplicates. The data points in panel **(G)** result from the quantification of toeprint signals of two independent experiments. Error bars represent the standard error. Further details are given in “Materials and Methods.”

To uncover the possible effects of cold-shock trans-acting factors on the translation of mRNAs with unfavorable secondary structures at low temperature, we first used a cell-free translation system containing crude S30 extracts (i.e., a bacterial content deprived from membrane debris) prepared from cells grown at 37°C (control) or exposed to 15°C for 120 min (cs extracts). The latter type of extract contains high levels of cold-shock proteins synthesized when cells reprogram their genetic expression after sensing the cold (Giuliodori, 2016). *In vitro* translation reactions were programmed with *cspA* mRNA, which acts as useful tool for studying structural transitions in RNA and the role of CS factors. After denaturation at 90°C, this transcript can be refolded in two different structures: the cold-structure, which exists only at a temperature below 20°C, and the 37°C-structure, which is stable when transferred at low temperatures after folding (Giuliodori et al., 2010). The cold-structure is competent in efficient recruitment of 30S ribosomal subunits to AUG initiation codon and presents only weak structures at the beginning of the coding sequence (Giuliodori et al., 2010; Figure 1A). Differently, in the 37°C-form both the SD and the initiation codon are partially occluded and a very stable structure (ΔG = −12.10; Giuliodori et al., 2010) is formed few nucleotides at the 3′ of the 30S mRNA channel of initiating ribosomes (Figure 1B).

The translational activities of the two *cspA* mRNA forms were tested with the two types of cell extracts at 15°C (Figure 1C). The data show that the cold-structure is efficiently translated with both control and cs extracts, while the 37°C-structure is efficiently translated only with the cs extract. This suggests that the intracellular milieu of cold-treated *E. coli* is enriched in factors that support the translation of the mRNA with the less favorable secondary structure. Since the most abundant protein in the cold-shock extract—CspA—is able to stimulate protein synthesis at low temperature (Giuliodori et al., 2004), we next investigated its role in translation. To this end, translation of the cold- and 37°C-forms of *cspA* mRNA was studied in the presence of increasing amounts of purified CspA using 70S ribosomes and post-ribosomal supernatant (S100) prepared from cells that were not exposed to low temperature (Figure 1D). The reactions were carried out at 15°C with the two forms of *cspA*, whereas at 37°C only the activity of the 37°C-structure of *cspA* mRNA could be probed, as the cold structure exists only at temperatures below 20°C (Giuliodori et al., 2010). Our results (Figure 1D) demonstrate that CspA promotes (> 2.5-fold) the translation of the less-favorable 37°C structure of *cspA* mRNA at low temperature, while it does not affect the translation of the other and more open *cspA* mRNA form. The effect is strongly dose-dependent, as the translation sharply increased when CspA concentration rises above 20–25 μM. Interestingly, this stimulatory activity of CspA is not observed at 37°C.

To investigate the mechanism by which CspA stimulates the translation process, we tested the effect of purified CspA on the recruitment of the mRNA conformers to the 30S subunit either in the presence (Figure 1E) or in the absence (Figure 1F) of IFs and the initiator fMet-tRNA_i_^Met^. We established that CspA does not assist the binding of its mRNA to the small ribosomal subunit, and we confirmed that the cold-form mRNA binds better (approximately 2.5-fold) to the 30S subunits than the 37°C-form mRNA (Giuliodori et al., 2010). Next, using filter binding (Supplementary Figure S1A) and toeprinting (Figure 1G; Supplementary Figure S1B), we probed the ability of CspA to promote the binding of fMet-tRNAiMet to the 30S subunits and the consequent formation of the active initiation complexes in the presence of the 37°C-form of *cspA* mRNA at low temperature. The toeprinting assay (Fechter et al., 2009) is based on the ability of the 30S translation initiation complex to stop cDNA synthesis by reverse transcriptase on the same template mRNA (Supplementary Figure S1B). The two experiments showed that the assembly of the initiation complex is insensitive to the addition of CspA.

### *CspA* assists the progression of the ribosome along the structured mRNA at low temperature

We next explored whether this protein could stimulate the translation elongation step in the cold. To this end, we have developed a test system based on the *E. coli* RelE toxin, which cleaves between the second and the third nucleotide of the mRNA codon in the ribosomal A site in the absence of the cognate A-site tRNA (Pedersen et al., 2003; Neubauer et al., 2009), in a so-called “RelE walking” experiment. The radiolabeled cold- and 37°C-forms of *cspA* mRNA were translated *in vitro* at 15°C using the PURE system (NEB)—a reconstituted system of the *E. coli* translation machinery with reduced concentration of charged asparagine tRNA (Asn-tRNA^Asn^; Inoue et al., 2001; Shimizu et al., 2001). At the end of the incubation, chloramphenicol and RelE were added to the reaction mixtures to stabilize the polysomes and to cut the mRNA at the codons in which the ribosomes were blocked due to the low content of Asn-tRNA^Asn^, respectively. Using polyacrylamide gel electrophoresis (PAGE), we monitored the extent of RelE cleavages on the three Asn triplets AAC (13th, 39th, and 66th codon) and on the first A-site codon after the AUG. This experiment provides new data concerning the fraction of ribosomes engaged in mRNA translation and the ribosomal progression along the transcript.

**Figure 2:**
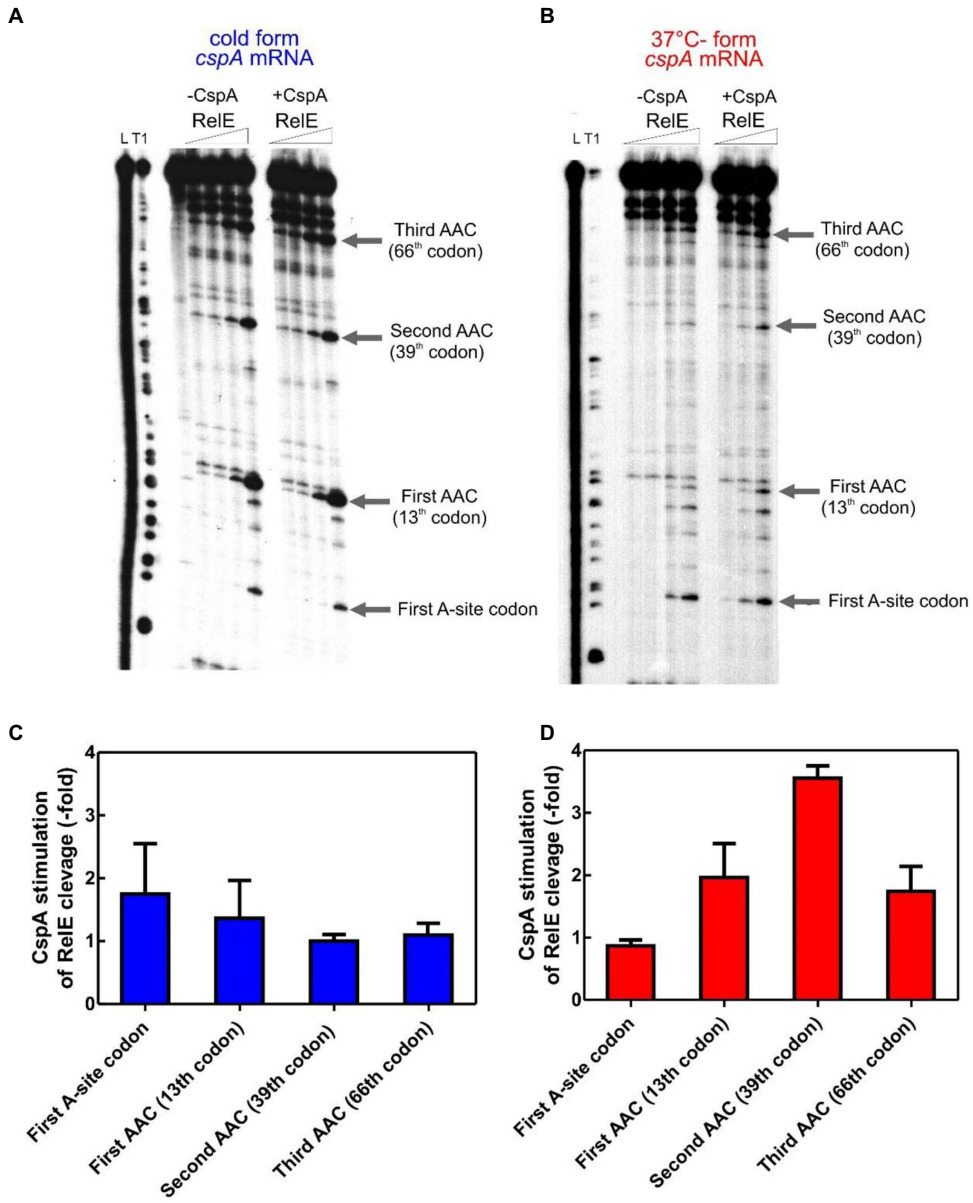
RelE walking experiment. ^32^P-labelled *cspA* mRNA, folded in the cold-structure **(A)** or in the 37°C-structure **(B)**, was used as templates for an *in vitro* translation assay with the PURE system and then cleaved with 0, 0.16, 0.72, and 1.44 μM of RelE. Lane L: alkaline ladder; lane T1: RNase T1 ladder. The first A-site codon (after the AUG initiation codon) and the AAC codons specifying Asn are indicated. Numbering is given according to the initiation codon. **(C)** and **(D)** show the effect of CspA (30 μM) on the intensity of RelE cleavages (normalized to the total radioactivity present in each lane) on the 15°C- and 37°C-forms, respectively. The three different RelE concentrations were used to calculate the observed fold change and the associated statistical variance (standard deviation).

Figure 2A shows that RelE cleaves extensively all Asn codons of the cold *cspA* mRNA form, independently of CspA, confirming that this conformer is adapted to be translated at low temperature. Interestingly, the intensities of the RelE cleavages detected with the 37°C-form of *cspA* mRNA (Figure 2B) are much weaker compared to those of the cold-form, the only exception being the cuts at the first A-site codon, which are comparable in the two forms of *cspA* mRNA. The data suggest that the number of ribosomes transiting along *cspA* mRNA and pausing at the asparagine codons is significantly lower in the case of the highly structured 37°C-form than in the cold-form of the mRNA. Notably, the addition of CspA to the translation system programmed with the 37°C-form causes intensification of the RelE cleavages, suggesting that the number of elongating ribosomes has enhanced (Figure 2B). Indeed, quantification of the gel bands (Figures 2C,D, normalized values) reveals that CspA induces on average a 2.5-fold increase of progression with the 37°C-form, a value very close to the observed stimulatory effect on translation (Figure 1D). The fact that CspA does not affect the rate of RelE cleavage of the cold-form of *cspA* mRNA (Figure 2C) excludes the possibility that CspA could influence directly the RelE activity. The fact that the 3′ termini of the two *cspA* conformers are identical (Giuliodori et al., 2010) excludes the possibility that the diverse RelE cleavage rates between the two forms could be promoted by a different rate of ribosome recycling. Finally, the RelE cuts at the A-site of the 70S initiation complex are similar in the absence and in the presence of CspA, thus ruling out the possibility that CspA could favor the occupancy of the A site by the aa-tRNA in the initiation phase. Here the signals corresponding to the translation initiation complex depends on the efficiency of formation of the complex, on the rate at which it transits to the elongation phase and on the availability of ribosomes to reinitiate after a translation round. It is therefore not surprising that although the efficiency of translation initiation is higher for the cold-form, the signals on the first A-site codon are similar in the 37°C-form.

Based on the RelE walking experiments, we propose that CspA promotes progression of the ribosomes on the highly structured 37°C-form during translation elongation at low temperature.

### Binding of *CspA* to *cspA* mRNA is responsible for translation stimulation

Isothermal Titration Calorimetry (ITC) is a powerful technique for studying interactions between native proteins and their RNA targets (Krimmer and Klebe, 2015). We used this approach to probe the possible interaction of CspA with the 70S ribosome (Supplementary Figure S2A), individual 30S (Supplementary Figure S2B), and 50S subunits (Supplementary Figure S2C) at 15°C, or 25°C and 35°C (Supplementary Figure S3). We fail to detect a specific interaction between CspA with either the 70S ribosome or the isolated subunits. The small variations observed are due to the heat released by the disassembly of CspA or ribosome aggregates in buffer upon dilution (Supplementary Figure S3). On the other hand, as expected, we detected the specific binding of initiation factor IF1 to the 30S subunit at low temperature (Supplementary Figure S2D) under comparable conditions (Kd = 806 nM). Notably, IF1 and CspA share impressive structural similarity (Gualerzi et al., 2011) and are both RNA binding proteins (Phadtare and Severinov, 2009); however, IF1 overexpression in *E. coli* does not suppress the defects of the *csp* quadruple deletion strain (Phadtare and Severinov, 2009).

CspA has been shown to bind RNA including the 5′-UTR of its own transcript (Jiang et al., 1997). Therefore, we investigated CspA-*cspA* mRNA interaction using Electrophoresis Mobility Shift Essay (EMSA). Given the large size (428 nts) of the full-length *cspA* transcript and the small mass of CspA (7.4 KDa), separation of such complexes constituted a technical challenge. Therefore, the analysis was done using a *cspA* mRNA fragment of 187 nts (*187cspA* RNA) consisting of the whole 5′ UTR plus 27 nts of the coding region. Importantly, this fragment adopts a secondary structure highly similar to that found in the full-length transcript at low temperature (Giuliodori et al., 2010). Complex formation with increasing concentrations of CspA is shown in in Supplementary Figures S4A,B. Below 80 μM of CspA, a minor gel retardation is observed (indicated with a thin arrow). However, as the amount of CspA exceeds 80 μM, a super-shifted band appears (indicated with a thick arrow), whose mobility continues to decrease with increasing amounts of CspA. This result suggests that the *187cspA* RNA contains one or multiple binding sites for CspA that are progressively occupied as the concentration of the protein rises. The appearance of the super-shift supports the hypothesis of a cooperative binding to RNA by CspA (Jiang et al., 1997; Lopez and Makhatadze, 2000). Alternatively, the band with minor gel retardation observed at lower CspA concentration could be the *187cspA* RNA with altered migration due to CspA-induced RNA conformational changes, while the main shifted band would correspond to single CspA binding events occurring at higher CspA concentrations. These experiments demonstrate that CspA binds to its mRNA at both low (Supplementary Figure S4A) and high temperatures (Supplementary Figure S4B) and confirm that this protein can bind also structured RNA molecules, although in this case the complexes are formed only at high protein concentrations.

The details of the CspA:*cspA* mRNA interaction at 15°C were then dissected using three different approaches: (i) UV-induced cross-linking and (ii) enzymatic probing, and Fe-EDTA footprinting. UV-induced cross linking is a technique which relies on the photoreaction between spatially close nucleotide bases (pyrimidines>purines) and specific amino acids at 254 nm, useful to localize on the RNA the protein binding sites. In addition, the RNA structure and its ligand-induced conformational change can be probed using structure-specific RNases (see below). Finally, Fe-EDTA footprinting exploits hydroxyl radicals which cleave the RNA backbone, regardless of secondary structures; the regions exposed/protected from hydrolysis are those involved in molecular interaction with the ligand.

**Figure 3:**
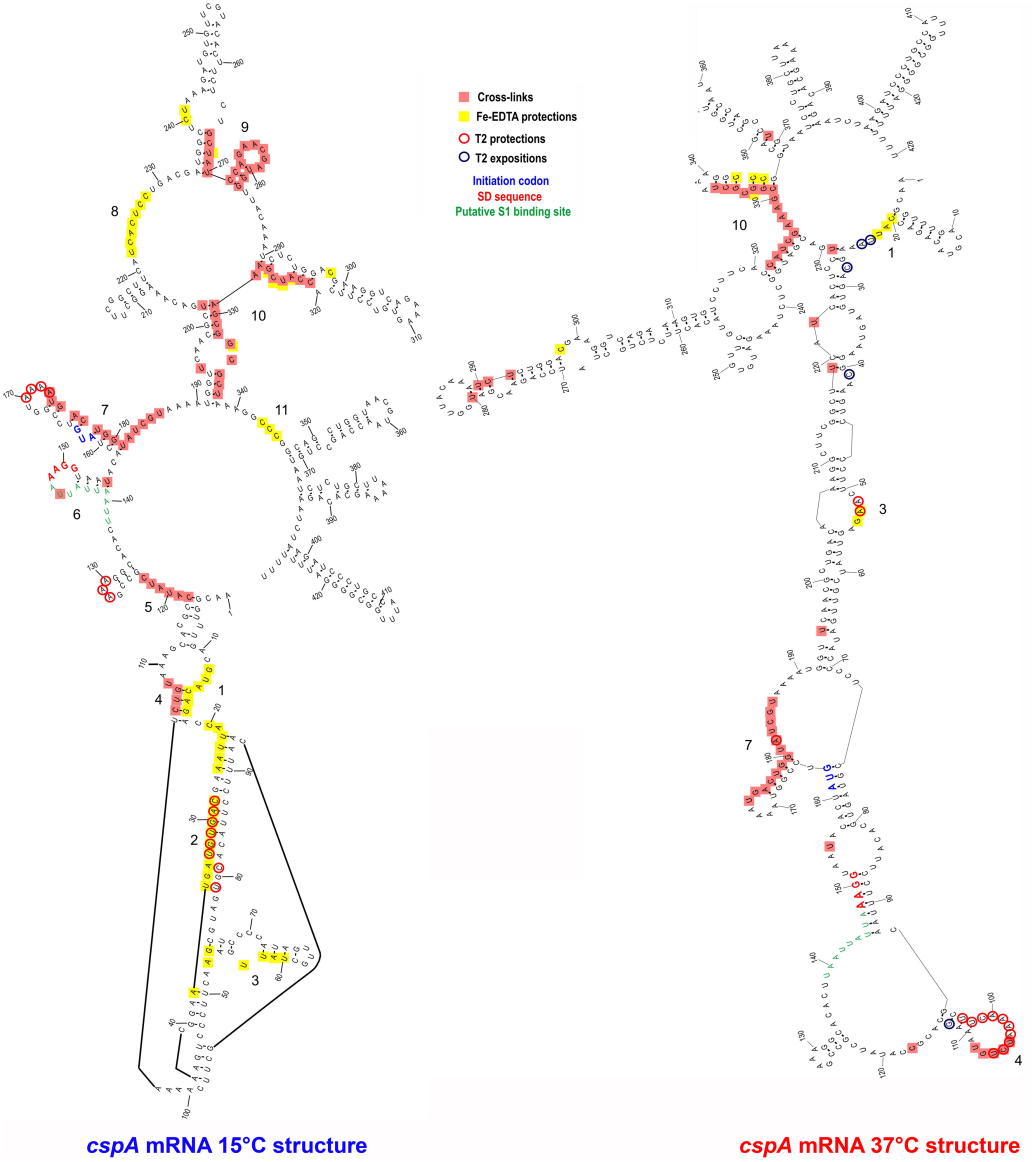
*CspA* bindig sites identified on both *cspA* mRNA forms. The footprinted/crosslinked positions reported on the structural model derive from the probing/cross-linking/Fe-EDTA experiments performed at 15°C using the *cspA* mRNA folded in the cold-conformation or in the 37°C-conformation. The SD sequence (red), the start codon (blue), and the putative S1-binding site (green) are indicated. The secondary structure model of *cspA* mRNA is taken from Giuliodori et al. (2010).

The resulting cross-link and footprint patterns are reported in the structure models of the cold-form and the 37°C-form (Figure 3) of *cspA* mRNA, while the electrophoretic analyses are shown in Supplementary Figures S5–S7. In the cold-form, we identified 11 main sites, which were either protected or cross-linked to CspA. Overall, the CspA sites are mainly positioned in apical or internal loops, extending also into the adjacent helices. Most of these sites are rather large, especially sites 1, 7, 9, and 10, which are located at positions 12–36, 170–186, 266–281, and 321 to 337, respectively. Notably, the CspA-induced cross-links at sites 7, 9 and 10, which also overlap with CspA induced protections against enzymatic cleavages or FE-EDTA, are particularly strong. Probing the 37°C-form of *cspA* mRNA bound to CspA shows significantly different patterns as compared to the cold-form. Only 5 of the 11 sites present in the cold-form had a counterpart in the 37°C-form, namely sites 1, 3, 4, 7, and 10, while the other regions became insensitive to CspA (sites 2, 5, 6, 8, 9, and 11). Furthermore, the binding sites were shorter as compared to the cold-form, with the exception of site 7 at the beginning of the coding region, which remained quite extended. Finally, the cross-links were overall less intense and much more dependent upon CspA concentration than in the case of the cold-form.

The above-described differences can be likely attributed to the more compact structure of the 37°C form, characterized by a long helix interrupted by several internal loops and bulged bases formed by the interaction between the 5′ UTR and part of the coding region (nucleotides C232 to G326). This closed conformation is further stabilized at low temperature (15°C) at which the probing experiments were performed (Giuliodori et al., 2010). Indeed, the reduced binding of CspA to the 37°C-form mRNA is not surprising considering the preference of Csp proteins for single stranded nucleic acids. Most likely, CspA needs unstructured regions for the initial contacts with the target RNA.

### Binding of *CspA* to a short *cspA* mRNA fragment affects the mRNA conformation

**Figure 4:**
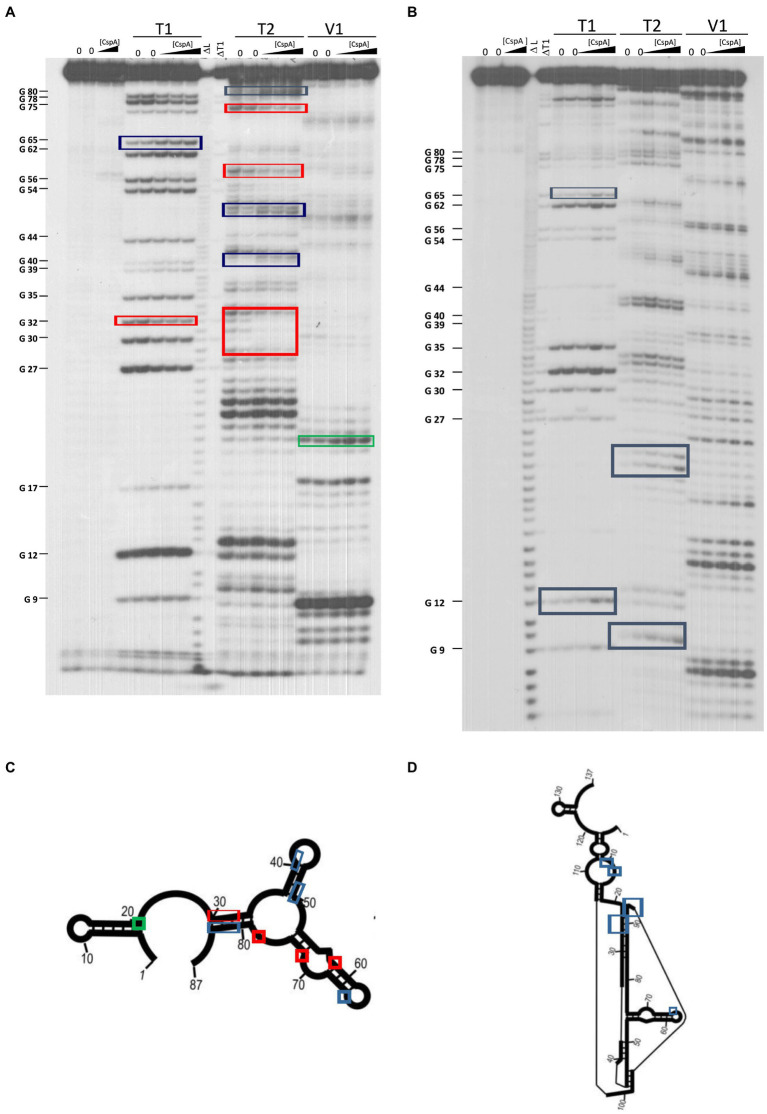
Footprinting experiments of fragments *87cspA* and *137cspA* RNAs. **(A)** Short electrophoretic migration of the fragments generated by RNase T1 (T1), RNase T2 (T2), or RNase V1 (V1) digestion of 5′-end [^32^P]-labelled **(A)** *87cspA* RNA and **(B)** *137cspA* RNA. The experiments were carried out in the absence (lanes 0) or in the presence of 51, 81, and 105 μM of CspA (increasing concentrations are indicated with a triangle). Lane ΔT: RNase T1 cleavages under denaturing conditions; lane ΔL: alkaline ladder. The red and blue boxes indicate the positions protected or exposed by CspA, respectively, while the green boxes indicate the V1 cuts enhanced by CspA. The same positions are reported on the schematic structural model (Giuliodori et al., 2010) of the *87cspA* RNA **(C)** or of the *137cspA* RNA **(D)**.

The secondary structure of three *cspA* mRNA fragments of increasing length (i.e., 87, 137, and 187 nts) from the transcriptional start site was previously analyzed (Giuliodori et al., 2010). These *cspA* mRNA fragments were designed as representative of RNA folding intermediates occurring during transcription. Their structures do not vary with temperature, and the *137cspA* and *187cspA* fragments adopt similar folding as in the full length cold-form of *cspA* mRNA. To investigate the role played by CspA on the initial mRNA folding process, we have analyzed the footprint of CspA on the 87cspA, 137cspA, and *187cspA* RNA fragments using RNase V1 (specific of double-stranded regions), RNase T1 (specific of unpaired guanine), and RNase T2 (specific of unpaired A > U > C). Binding of CspA to the short 87cspA RNA significantly affects the RNase cleavage pattern at a concentration of CspA >50 μM (Figures 4A,C). For instance, protections against RNase T2 were observed between A29 and U33, and at positions U58 and U76; concomitantly enhanced RNase cleavages were found at positions C41, C50, A51, G65, and C81-A82. On the other hand, the addition of high concentrations of CspA had only minor effects on the structure of 137cspA RNA (Figures 4B,D); Supplementary Figure S8). For instance, the CspA-dependent protections in the region A29 and U33 of 87cspA RNA were no longer observed. As for the *187cspA* RNA (Supplementary Figure S9), enzymatic probing confirms CspA binding observed with EMSA (Supplementary Figure S4) and indicates only local structural CspA-induced changes with this *cspA* fragment.

Therefore, overall these data suggest that CspA might have different functional impacts *in vivo* that will depend on many factors including kinetics of RNA transcription and folding.

### *CspA* preferentially binds to short RNA sequences containing YYR motif.

Inspection of all sites protected by CspA revealed that 11 out of 15 of these regions comprise an YYR (pyrimidine-pyrimidine-purine) motif. Multiple alignments were performed using the YYR motif as the reference sequence (Supplementary Figure S10A). Although the motif is highly degenerated, the YYR motif seems not to be followed by a G two positions downstream from the R. The degree of specificity of CspA for the identified sequence features was then tested by tryptophan fluorescence titration experiments. Intrinsic protein fluorescence originating from excited tryptophans (Trp) is highly sensitive to its local environment and can be used as a reporter group for monitoring the binding of ligands, such as RNAs. We have used an RNA oligonucleotide (Oligo1: 5′-AACUGGUA-3′) whose sequence reflected the conserved positions as shown in Supplementary Figure S10B. The experiment was also performed with 5 other RNA oligos in which each one of the bases located in the central positions of Oligo1 was individually replaced by A. In addition, a poly-A oligo was also used. The data (Supplementary Figure S10C; Table 1) show that the single nucleotide changes caused only small variations of the dissociation constant (K_D_ around 1 μM), with the exception of oligo 6 (G replaced by A at position 6), which produced a 5-fold increase of the K_D_. A similar decrease of affinity was observed with the poly-A oligonucleotide. This result is in agreement with earlier works (Jiang et al., 1997; Lopez and Makhatadze, 2000) reporting the K_D_ for the CspA-RNA complex in the μM range.

**Table 1:** Equilibrium dissociation constants of the CspA:RNA oligonucleotide complexes determined by tryptophane fluorescence titration.

Oligonucleotide	Sequence 5′ to 3′	*K*_D_, μM
Oligo 1	AACUGGUA	1.24 ± 0.08
Oligo 2	AAAUGGUA	2.30 ± 0.13
Oligo 3	AACAGGUA	1.24 ± 0.05
Oligo 4	AACUAGUA	1.03 ± 0.06
Oligo 5	AACUGAUA	4.86 ± 0.24
Oligo 6	AACUGGAA	2.08 ± 0.14
Oligo 7	AAAAAAAAA	5.13 ± 1.15

Because there is a high degree of sequence and structure similarity between *E. coli* CspA and its *B. subtilis* orthologue CspB, we produced a homology model of CspA in complex with Oligo 1 using the available 3D structure of a CspB-RNA complex (Sachs et al., 2012). This model (Supplementary Figure S10D) indicates the path that the RNA could take on CspA. The RNA backbone wraps around Lys16 and is maintained by Lys 60 on the surface of a cleft containing aromatic residues important for stacking interactions with the bases. The cleft allows fitting of the YYR core motif, while other combinations of trinucleotides like RRR would be less easily recognized due to the steric hindrance of the surrounding residues, especially Lys60 and Lys28. From the model, the aromatic sidechains of Phe31, Phe20, and Trp11, would stack with C3, U4, and G5, respectively (Supplementary Figure S10D), while G6 is stacked on G5. Hence, RNA binding is dominated by stacking interactions between the YYR motif and the aromatic protein sidechains of Phe31, Phe20, and Trp11. Furthermore, the purine downstream from the YYR motif can strengthen the stacking of the side chain of Trp11 with G5, while the nucleotide (A/U) upstream the core motif can stack with the sidechain of His33, further stabilizing the protein-RNA interaction. Taken together, our data support that the YYR might be the preferred seed sequence to initiate binding. This RNA recognition mechanism could be common to other CspA paralogues and orthologues. In fact, all Csp proteins contain two nucleic acid-binding sequence motifs, RNP1 (including Lys16 and Phe20) and RNP2 (including Lys28 and Phe31), which are also present in the eukaryotic gene-regulatory “Y-box” proteins. Multiple sequence alignments (see Ermolenko and Makhatadze, 2002) showed that the amino acid residues of these motifs are highly conserved. Trp11 and Lys60, albeit outside of these two motifs, are also highly conserved in bacterial Csps and in some human Y-box proteins. In *E. coli*, the most divergent Csp proteins are CspF and CspH, in which several of these important residues are not conserved.

### *CspA* promotes translation of numerous CS and non-CS mRNAs at low temperature

**Figure 5:**
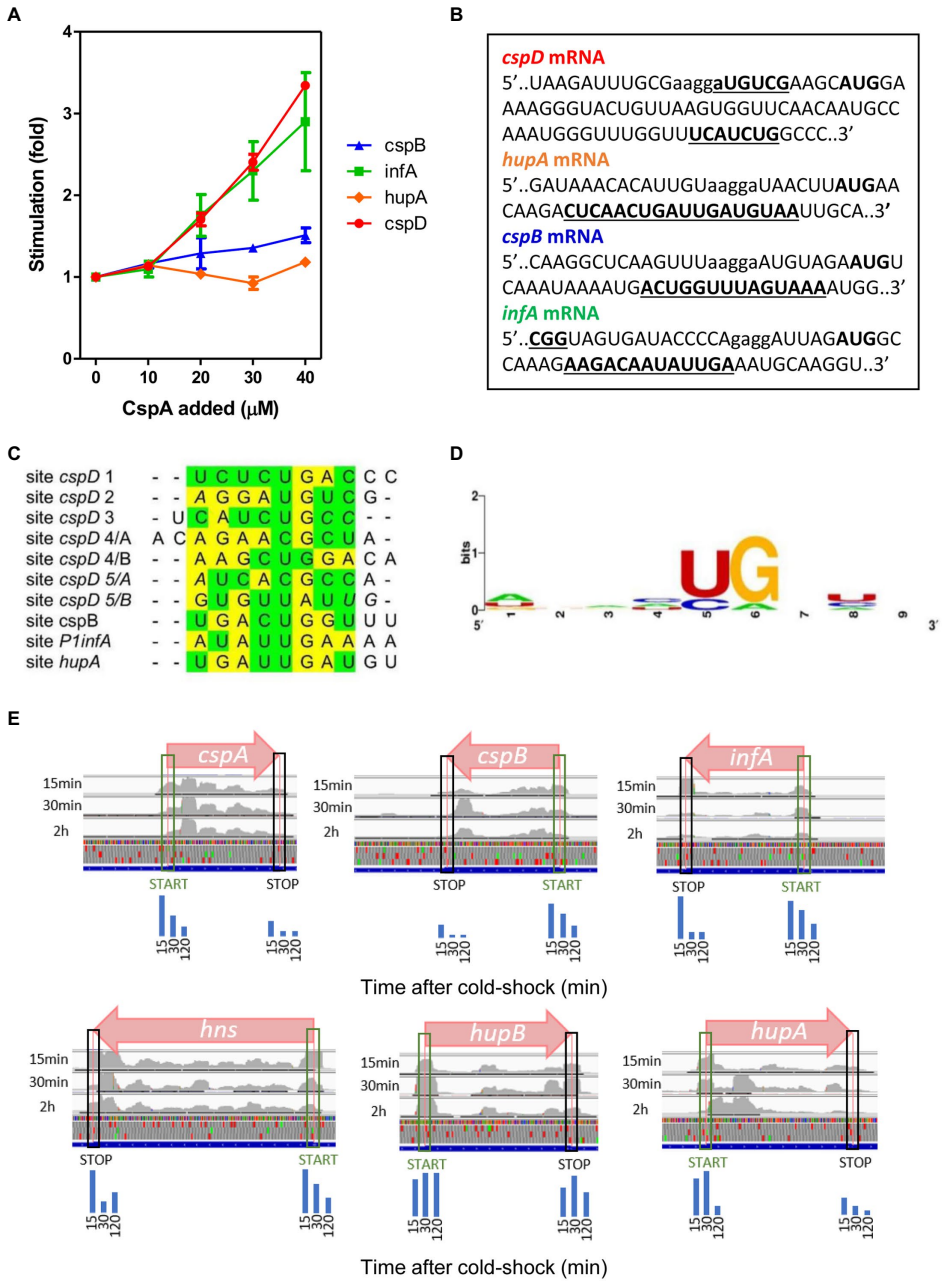
CspA binding to various mRNAs and functional effects. **(A)** *In vitro* translation at 15°C performed with control 70S and S100 in the presence of *P1infA* mRNA (green squares), *cspD* mRNA (red circles), *cspB* mRNA (blue triangles), *hupA* mRNA (orange diamonds) and the indicated amounts of purified CspA. The data points are the average of two independent experiments and the error bars represent the standard error. **(B)** CspA binding sites (bold underlined) identified around the Translation Initiation Region (TIR) of the indicated mRNAs by crosslinking experiments performed at 15°C. SD sequence and start codon are indicated in lower case and bold, respectively. **(C)** Manual multiple alignment of the sites cross-linked/footprinted by CspA on the indicated mRNAs. Since CspB, the CspA-homolog of *Bacillus subtilis*, recognizes a sequence of 6–7 nts (Lopez and Makhatadze, 2000; Sachs et al., 2012), we hypothesized that the sites in which the cross-links/footprints were particularly extended (> 12 nts) could be the results of the binding of two adjacent CspA molecules. For this reason, we divided these extended sites into two sub-sequences of comparable length, named A and B, which were used to build the alignment. The yellow and green highlighting indicates purines and pyrimidine, respectively. The bases in italics are adjacent to those crosslinked by CspA. **(D)** Logo representation of the CspA binding preference derived from the alignment shown in panel C and generated with WebLogo (Crooks et al., 2004). **(E)** Coverage tracks of aligned reads from ribosome protected fragments derived from Zhang et al. (2018) visualized by IGV genome browser. Normalized ribosome densities are displayed at initiation and stop codons of different genes. Blue bars represent the quantization of peak densities at the start and stop codons at the three time points. From left to right, 15 min, 30 min, and 2 h, respectively. Normalized coverage tracks for *cspA*, *cspB*, *infA*, and *hns* mRNAs show peaks on start codons which progressively decrease during cold acclimation. Normalized coverage tracks for *hupA* and *hupB* mRNAs do not show the same trend and their initiation peaks remain relatively high even after several minutes of cold acclimation. *CspD* mRNA is not expressed under these conditions.

To gain additional insight into the CspA properties we tested the effect of CspA on the *in vitro* translation of mRNAs other than *cspA*. Two classes of mRNAs were selected to carry out this analysis: (i) the cold-shock transcripts *cspB* and *P1infA*, the former belonging to the *E. coli csp* gene family (Yamanaka et al., 1998) and the latter originating from the P1 promoter of *infA* and encoding Initiation Factor 1 (IF1; Giangrossi et al., 2007); and (ii) the non-cold-shock transcripts *hupA* and *cspD*, encoding the α-subunit of the nucleoid associated protein HU (Giangrossi et al., 2002) and protein CspD (Yamanaka and Inouye, 1997), respectively. As shown in Figure 5A, the translation of *infA* and *cspD* mRNAs is strongly stimulated by CspA (3–4 fold), that of *cspB* mRNA is moderately enhanced, while *hupA* mRNA translation is insensitive to CspA addition. This result confirms that CspA is able to stimulate the translation at low temperature of various transcripts other than its own mRNA (Giuliodori et al., 2004), but also indicates that this activity cannot be generalized.

The existence of the CspA-dependent translational stimulation of the tested mRNAs (*infA*, *cspD*, *cspB*) raises the question as to whether these transcripts were directly recognized by CspA. To address this issue, we mapped the possible interactions between CspA and our selected mRNAs by UV-induced crosslinking experiments at 15°C (Figure 5B; Supplementary Figure S11). Notably, there appears to be a binding site common to all tested mRNAs – apart from *cspD* mRNA. The average length of the binding site is of 14 nts and is located between 9–12 nts downstream from the G of the translation initiation codon (Figure 3; Supplementary Figures S5, S11). In the case of *cspD* mRNA, multiple cross-links were present along the entire mRNA (Supplementary Figure S11). In the region near the AUG codon, a cross-link of moderate intensity is observed in the SD region while a more intense one is located between the 17th and the 19th codons of the coding region. The YYR motif was found also in the regions cross-linked with CspA in these mRNAs. The multiple alignments built using the YYR motif as the reference sequence (Figure 5C) produced a Logo similar to that generated using the binding sites on *cspA* mRNA (Figure 5D).

Zhang et al. (2018) have shown by ribosome profiling that CspA contributes to support translation recovery of the other genes during cold-shock by analyzing three time points after temperature down-shift (15 min, 30 min, and 120 min). Ribosome profiling relies on the sequencing of mRNA fragments protected by the translating ribosomes and analogously to our *in vitro* RelE walking assay, allows to map ribosome pausing or stalling sites, which are evinced by peaks when the data are visualized on a genome browser. To understand if we could observe *in vivo* the proposed stimulation of translation progression from initiation to elongation operated by CspA, we have used the data from Zhang et al. (2018) (see Material and Methods) to look for stalling sites on different mRNAs. Since CspA accumulates during the acclimation phase, we would expect to see peaks at the initiation codons in the early time points (15 min) progressively decreasing at later time (30 and 120 min). Interestingly, in the cell subjected to cold shock the peaks at the initiation codons of the mRNAs tested in this, as well as in a previous work (Giuliodori et al., 2004), progressively decrease with the time (Figure 5E), confirming the possibility that our proposed model for CspA stimulation of translation could take place *in vivo* on different mRNAs.

## Discussion

### The CspA paradox

Our data demonstrate that the binding of CspA to mRNA is not always accompanied with an effect on translation. This was particularly well illustrated with *cspA* mRNA: despite the extensive binding of CspA to the cold-form of *cspA* mRNA, translation of this structure is not stimulated, whereas the translation of the 37°C-form is enhanced by CspA although CspA binding is less efficient. How can this apparent paradox be explained?

Immediately after cold-shock CspA becomes a very abundant protein (Brandi et al., 1999), and it is estimated to be bound in several copies to cellular mRNAs (Ermolenko and Makhatadze, 2002). CspA was shown to bind its own mRNA (Jiang et al., 1997) and to act as an RNA chaperone (Jiang et al., 1997; Rennella et al., 2017; Zhang et al., 2018). In this work, we demonstrated that CspA is able to recognize in its mRNA short and degenerated sequences mostly located in single stranded regions, including internal and apical loops. Furthermore, we showed that CspA stimulates the translation at low temperature from its 37°C-form mRNA, which adopts a large and irregular hairpin structure sequestrating the SD sequence (Figure 3). This CspA-dependent translational stimulation is observed with other mRNAs. In all tested mRNAs, with the exception of *cspD*, we identified a cross-link positioned between 9–12 nts from the initiation triplet. We propose that this region of mRNAs could be a preferential CspA binding site since it does not usually adopt secondary structures (Del Campo et al., 2015). In spite of this interaction, our results demonstrate that CspA enhances the translation of only some of the mRNAs that it is able to bind. Our functional experiments performed with *cspA* mRNA demonstrate that the translational stimulation affects elongation rather than initiation. Particularly, the RelE-walking experiment indicates that this activity consists in facilitating ribosome progression on the mRNA at low temperature.

During translation the ribosome is able to melt secondary structures of the mRNA thanks to the helicase activity of S3, S4 and S5 proteins (Qu et al., 2011). It is very likely that this activity could be partly impaired by the low temperature, which is known to stabilize base pairing interactions, making it harder for the ribosome to melt the secondary structures (Liu et al., 2014). The presence of CspA on the mRNA could be useful to facilitate ribosome progression either by destabilizing some positions and/or by preventing the re-formation of the secondary structures after the first elongating ribosome has unwound them.

**Figure 6:**
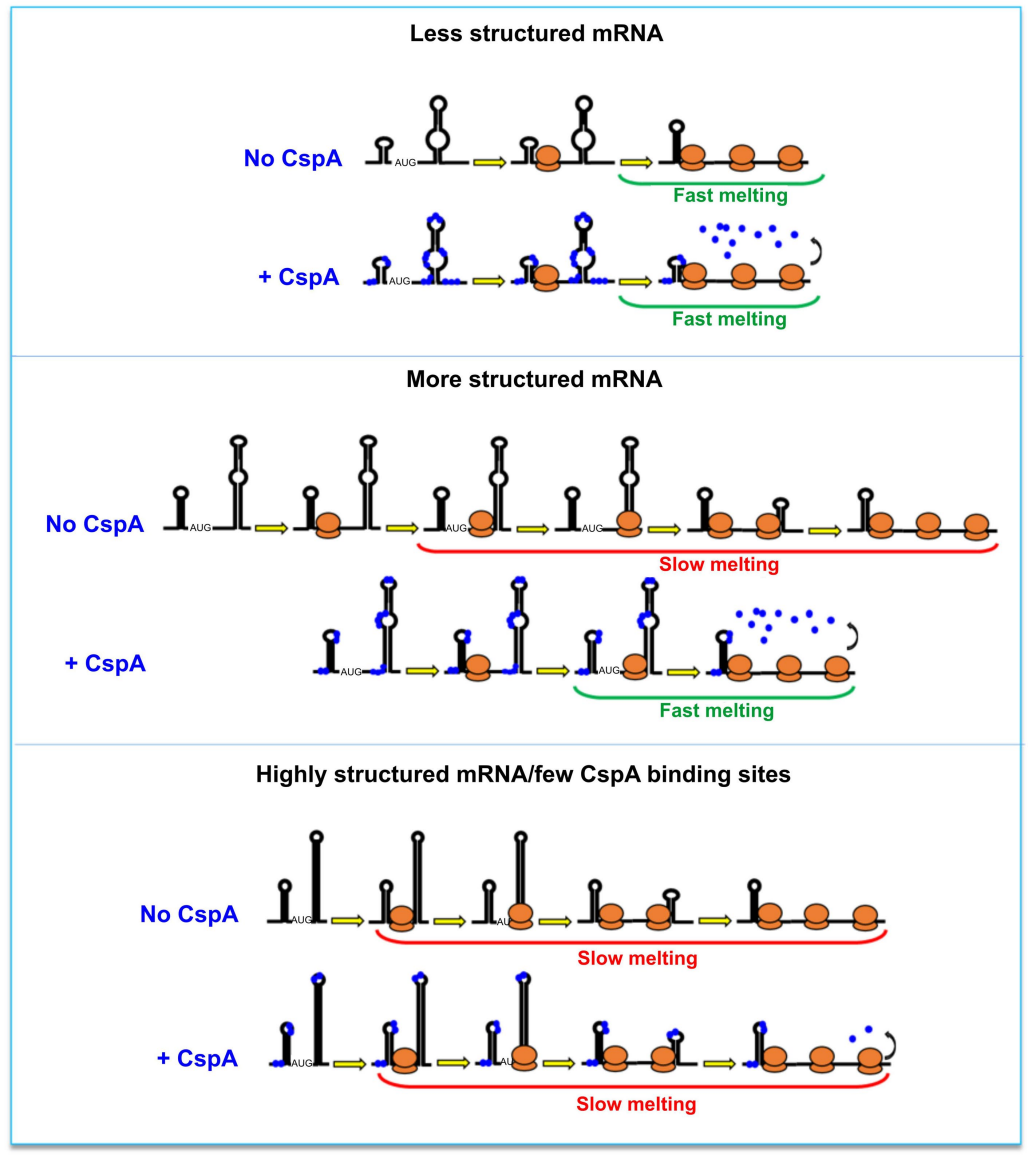
The CspA paradox. CspA would ensure ribosome progression by maintaining the bound regions unstructured and/or destabilizing the helices when ribosomes translate along the mRNA (middle panel). This effect will not be seen with mRNAs attaining an open conformation compatible with translation at low temperature (top panel), or in the case of mRNAs that are either structured or containing few sites suitable for CspA binding (bottom panel). A possible model postulates that the destabilization of mRNA structures by CspA will facilitate ribosome progression. In another model, the amount of the mRNA-bound CspA increases as the translating ribosomes open up the mRNA structures.

Based on our data, two mechanistic models, not mutually exclusive, can co-exist. The first model builds on the CspA RNA chaperone activity (Rennella et al., 2017). The model proposed by Renella and colleagues is based on kinetics and thermodynamics analyses obtained by real-time NMR experiments. It outlines how CspA is able to recognize loops, bulges or other single-strand regions on RNA and from this first interaction site can induce weakening of the base-pair interactions of nearby RNA structures. This ultimately would lead to the rearrangement of the bound RNA, which could establish new base-pair interactions and reach a thermodynamically more stable state, adopting a new structure. Therefore, in the presence of translating ribosomes, the destabilization of mRNA structures will transiently increase the proportion of single-stranded or metastable regions thus facilitating ribosome progression. In the second model, the amount of the mRNA-bound CspA increases as the translating ribosomes open up the mRNA structures. In this case, stimulation during translation would not depend on the amount of CspA pre-bound to the mRNA but rather on the capacity of CspA to rapidly bind (or re-bind) the regions melted by the passage of the first ribosome and keep them single-stranded, thus facilitating polysome formation. This RNA chaperone activity has been dubbed “overcrowding” (Cristofari and Darlix, 2002). Both models would predict an easy displacement of CspA molecules as the ribosomes transit on the mRNA interaction sites and can explain the “CspA paradox” (Figure 6). In fact, ribosome progression would be stimulated by CspA only with structured mRNAs whose conformational state is stabilized by the low temperature, while the effect will not be seen with mRNAs carrying a more open conformation, intrinsically suitable for translation at low temperature. It can thus be predicted that translation of mRNAs that are too structured and that contain too few sites for CspA binding would be little stimulated by CspA.

### Stimulation of *cspA* mRNA translation is one of the first tasks of CspA during cold acclimation.

At the beginning of the acclimation phase, both CspA and its mRNA are already present and the translation activation could be rapidly achieved through their interaction. Indeed, immediately after the cold stress the *cspA* mRNA transcribed at 37°C is stabilized 100 times, with its half-life increasing from 12 s to 20 min (Goldenberg et al., 1996; Fang et al., 1997). CspA protein is also known to be abundant at 37°C during early exponential growth, when its concentration reaches up to 50 μM (Brandi et al., 1999). Under these conditions the cold-shock induction of *cspA* is rather low (only about 3-fold) and at the onset of the cold stress the cells use predominantly *cspA* mRNA and CspA protein already synthetized at 37°C. Therefore, the capacity of CspA to favor the translation of its mRNA folded in the 37°C-conformation in the cold can speed up the accumulation of more *cspA* product, in a positive auto-regulatory loop. Our data suggest that the translational stimulation by CspA should take place at concentrations ≥25 μM (Figure 1D). In addition, probing/footprinting experiments suggested that CspA does not produce important conformational changes on the full length pre-folded *cspA* mRNA, even at concentrations >100 μM. However, CspA seems to affect the conformation of a *cspA* mRNA fragment corresponding to the first 87 nts. Therefore, CspA could play different roles in the cell depending on its expression level and/or the RNA structure stability, thus influencing the co-transcriptional folding process of its targets, *cspA* mRNA *in primis*. This hypothesis is supported by the work of Zhang et al. (2018), which have demonstrated that at a concentration of 100 μM, CspA can modulate the structure of its mRNA, as well as that of cspB, thereby making it more susceptible to degradation at the end of the acclimation phase.

In addition to CspA orthologues present in all bacterial taxa, multiple paralogues have wide evolutionary distribution (Graumann and Marahiel, 1996, 1998). Some of these paralogues carry out overlapping functions, as demonstrated by the fact that in *E. coli* four out of nine *csp* genes must be deleted to obtain a cold-sensitive phenotype and that the overexpression of any member of the *csp* family (except for *cspD*) suppresses the phenotype (Xia et al., 2001). It is also known that these small proteins act as anti-termination factors during transcription and can bind both ssDNA and RNA with different specificity. CspB, CspC and CspE display specificity for 5′-UUUUU-3′, 5′-AGGGAGGGA-3′ and 5′-AAAUUU-3′ sequences, respectively, with K_D_ values in the range of 1–10 μM, similar to that calculated for CspA. Thus, it is conceivable that during cold-shock various Csp could be bound to the mRNAs to regulate their transcription or modulate their structure, stability and translation, possibly in a concentration-dependent manner as in the case of CspA.

## Data availability statement

Publicly available datasets were analyzed in this study. This data can be found at: https://www.ncbi.nlm.nih.gov/geo/query/acc.cgi?acc=GSE103421.

## Author contributions

AMG and SM: conceptualization, methodology, and writing—original draft. AMG, MD, RB, RG, ES, and EE: investigation. AMG, MD, RB, RG, ES, VH, EE, and SM: writing—review and editing. AMG: visualization. AMG, EE, and SM: supervision and resources. All authors contributed to the article and approved the submitted version.

## Funding

The work was supported by the “Projet International de Coopération Scientifique” (PICS) No. PICS 5286 between France and Italy to SM. This work was supported by the Centre National de la Recherche Scientifique (CNRS) and by the French National Research Agency ANR (ANR-21-CE12-0030-01 to SM, ANR-10-LABX-0036 NETRNA). This work of the Interdisciplinary Thematic Institute IMCBio, as part of the ITI 2021–2028 program of the University of Strasbourg, CNRS and Inserm, was supported by IdEx Unistra (ANR-10-IDEX-0002), by SFRI-STRAT’US project (20-SFRI-0012), and EUR IMCBio (IMCBio ANR-17-EURE-0023) under the framework of the France 2030 Program. VH was supported by the Swedish Research Council (Vetenskapsrådet) grants (2021-01146), Cancerfonden (20 0872 Pj), and the Knut and Alice Wallenberg Foundation (2020-0037).

## Conflict of interest

The authors declare that the research was conducted in the absence of any commercial or financial relationships that could be construed as a potential conflict of interest.

The reviewer RE declared a shared affiliation with the author VH to the handling editor at the time of review.

## Publisher’s note

All claims expressed in this article are solely those of the authors and do not necessarily represent those of their affiliated organizations, or those of the publisher, the editors and the reviewers. Any product that may be evaluated in this article, or claim that may be made by its manufacturer, is not guaranteed or endorsed by the publisher.
