# Supplementary material for: Escherichia coli CspA stimulates translation in the cold of its own mRNA by promoting ribosome progression
Source: Front Microbiol. 2023 Feb 9;14:1118329. doi: 10.3389/fmicb.2023.1118329 (PMC9947658; doi:10.3389/fmicb.2023.1118329)
Supplement: Supplementary file 1 [file Data_Sheet_1.PDF]

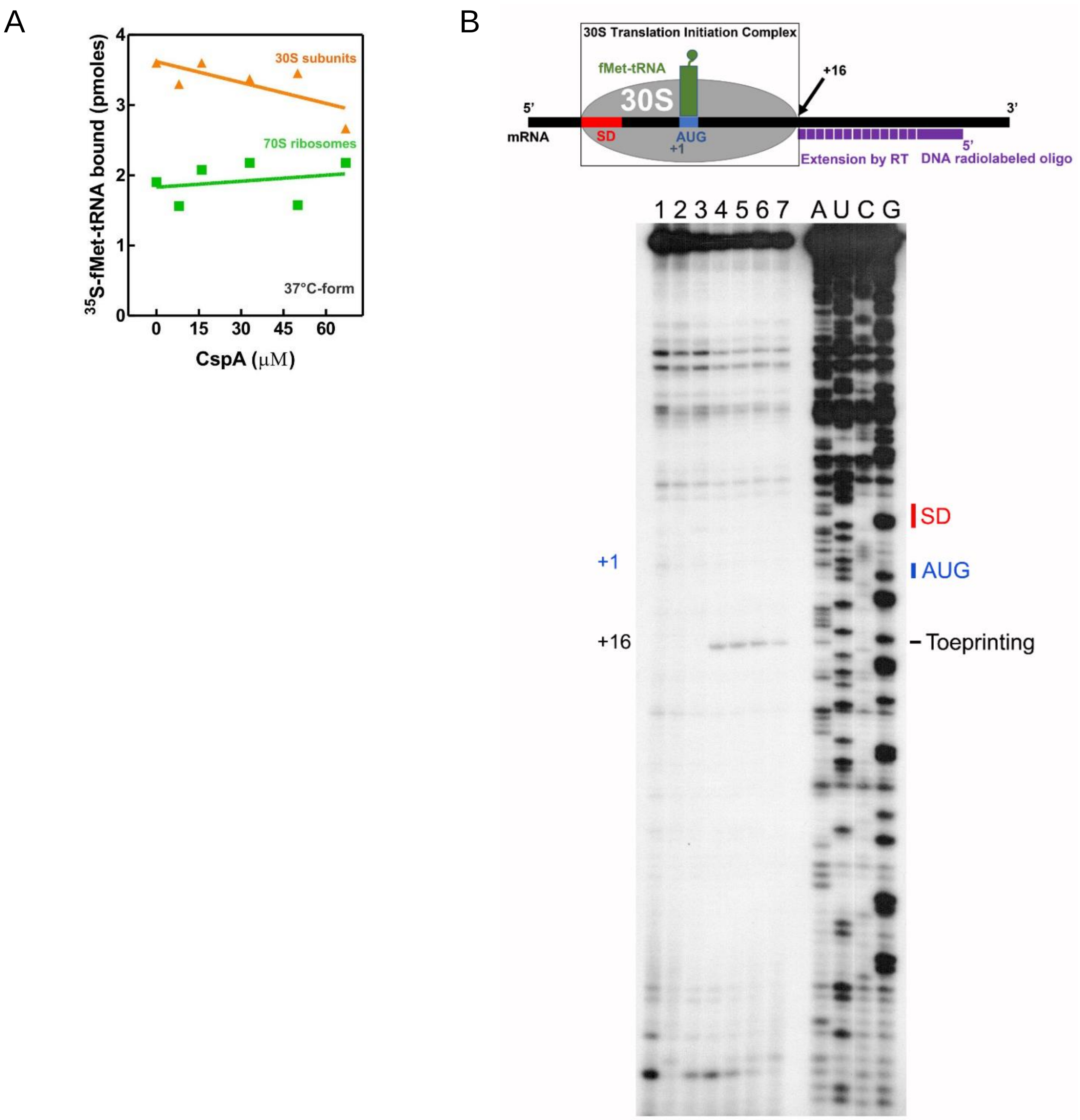

Figure S1. **Filter binding and toeprinting assays to monitor the effect of CspA on the binding of *cspA* mRNA to the ribosome and on the formation of the translation initiation complex, respectively.** (A) The binding of ^{35}S-fMet-tRNA$_i^{Met}$ to 30S subunits (green squares) and to 70S ribosomes (orange triangles) programmed with IFs and the 37°C-form of *cspA* mRNA by filter binding assay. (B) Effect of CspA on the formation of the initiation complex (30SIC) analyzed by toeprinting assays. The formation of a simplified 30SIC, composed of the 30S (grey oval in the scheme), the mRNA (black line), and the initiator fMet-tRNA (green) is observed by the signal at position +16 (+1 is the adenine of the start codon) obtained by a reverse transcriptase reaction carried on the same mRNA. Control lanes 1, 2 and 3: mRNA alone (lane 1), mRNA with 50 µM CspA (lane 2), mRNA with 30S ribosomal subunits (lane 3); lanes 4 to 7: 30SIC formed with either no CspA (lane 4), or with 15 µM (lane 5), 30 µM (lane 6), or 50 µM (lane 7) of CspA. Lanes A, U, C, G, sequencing ladders. Quantification of the toeprint was normalized according to the total amount of radioactivity (full-length extension and +16 product bands) using the ImageQuantTL software (GE Healthcare Life Sciences). The result of two independent toeprinting experiments is reported in main figure 1G.

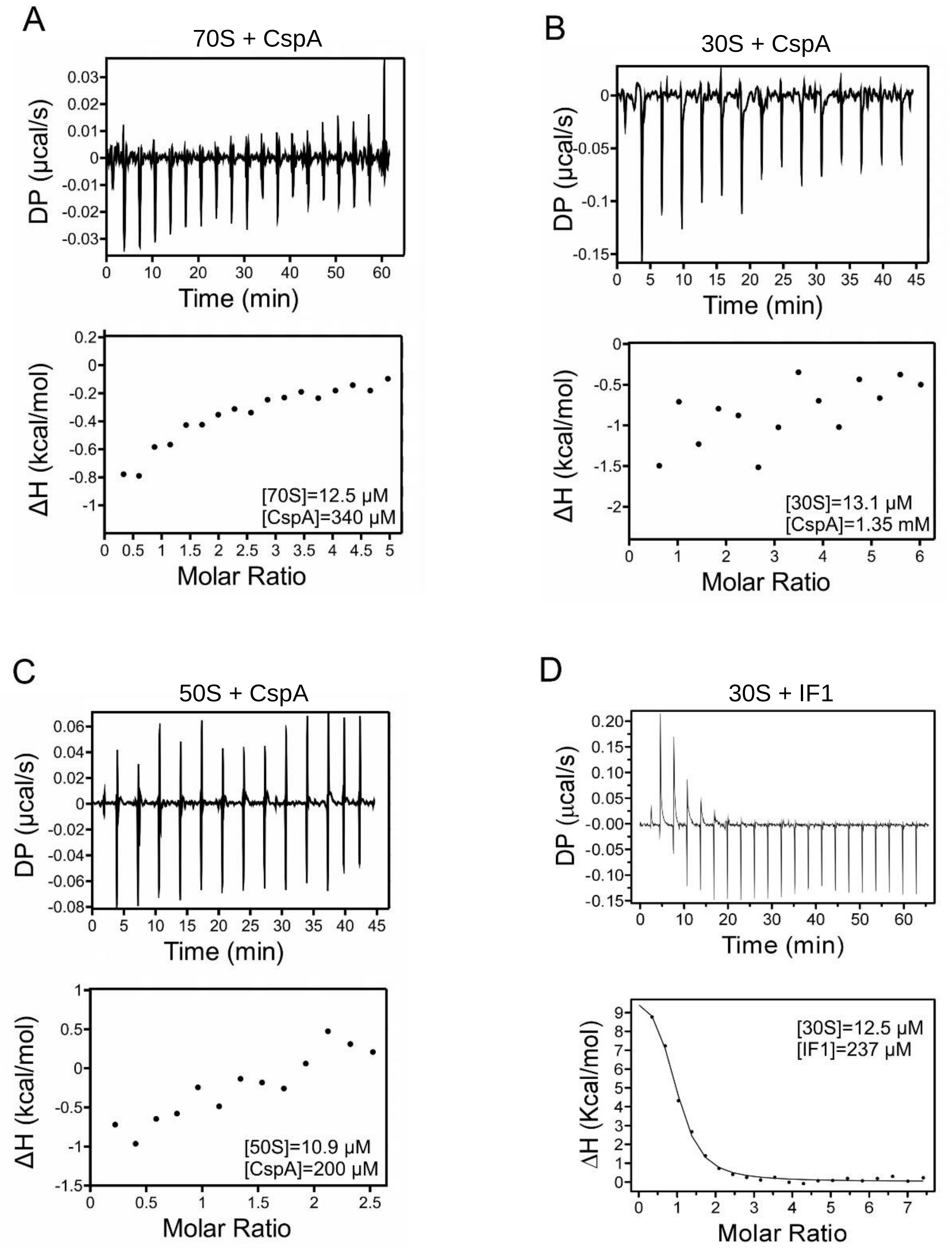

FIGURE S2. ITC analysis of CspA interaction with the 70S ribosomes and ribosomal subunits. Titration of *E. coli* ribosomes with CspA was studied at 15°C by sequential 2 µL injections of CspA in a cell containing 200 µL of (A) 70S ribosomes, (B) 30S subunits or (C) 50S subunits. (D) Control experiments were performed using IF1 and 30S subunits at the indicated concentrations.

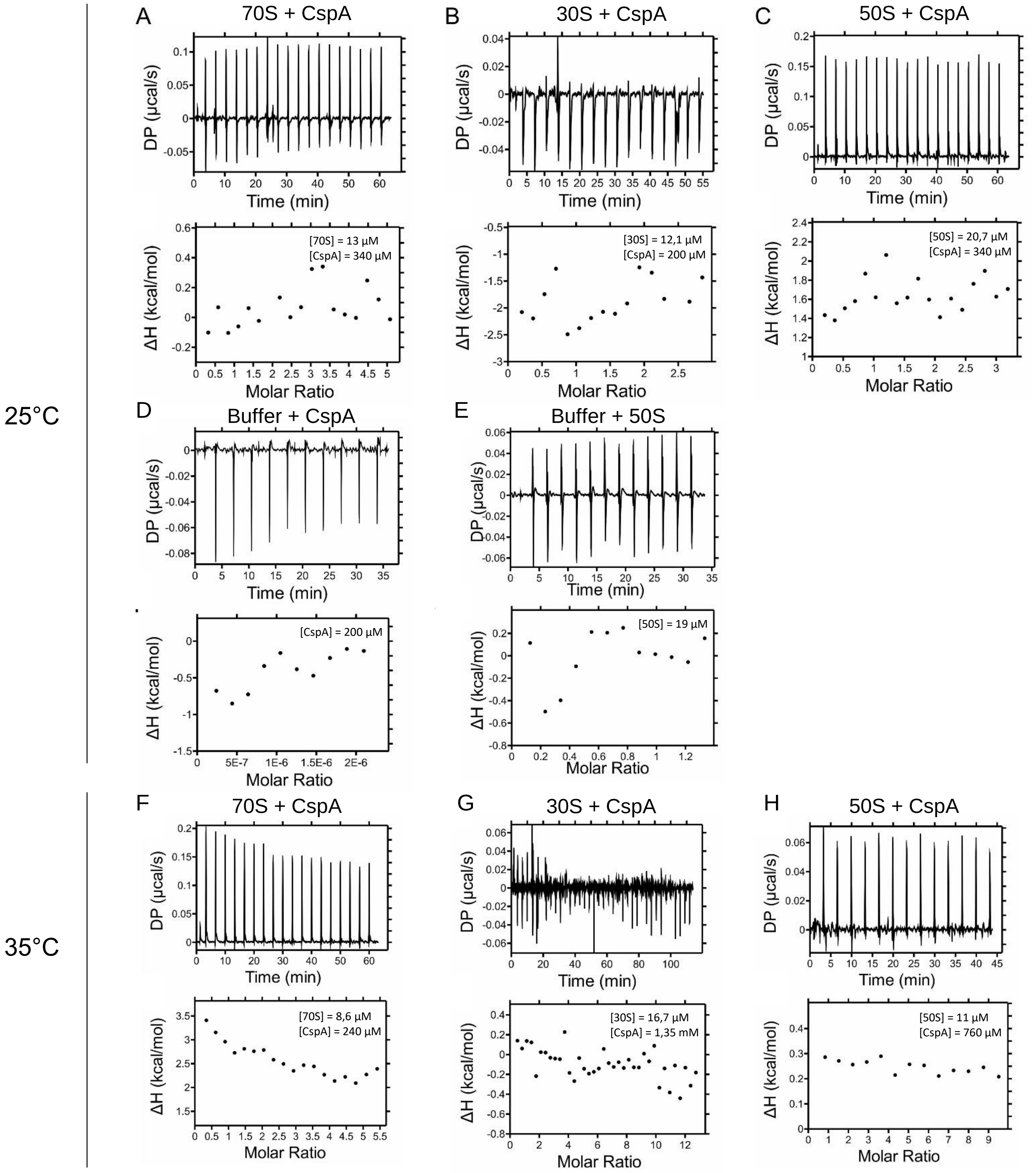

FIGURE S3. Binding of CspA to 70S ribosomes or ribosomal subunits. Titration of *E. coli* ribosomal particles with CspA was studied at 25°C (A-E) and 35 °C (F-H). Titration was carried out by consecutive 2 µL injections of CspA in a cell containing 200 µL of either 70S ribosomes (A, and F), 30S subunits (B and G) or 50S subunits (C and H). D and E are examples of signals of CspA and *E. coli* 50S subunits obtained upon dilution in ITC buffer at 25°C: in (D) 2 µL of CspA were repeatedly injected in the sample cell filled with ITC buffer, while in (E) 2 µL of ITC buffer were repetitively injected in the sample cell filled with 50S subunits.

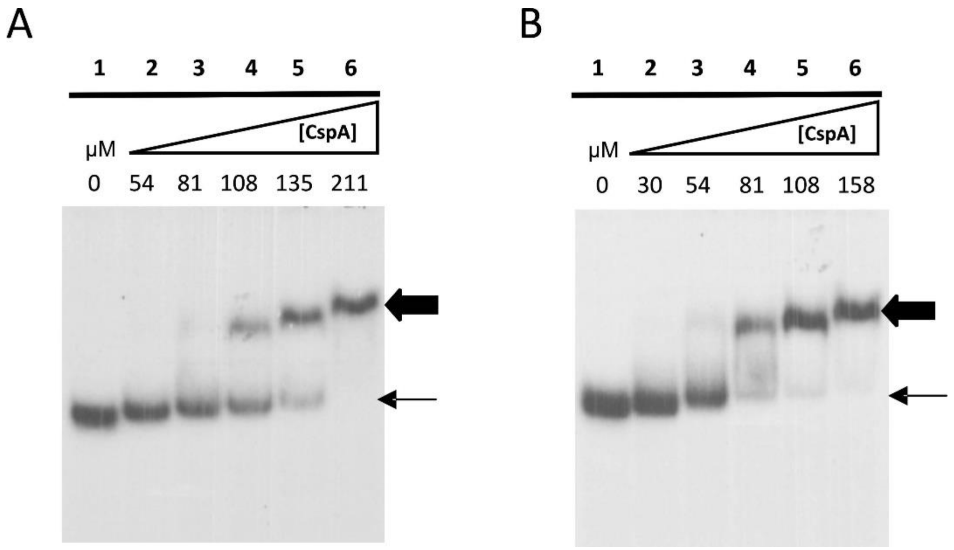

FIGURE S4. **CspA-*187cspA* RNA interaction studied by EMSA**. The binding of CspA to ^{32}P-labeled *187cspA* RNA performed at 20°C (A) or at 37°C (B) was analyzed in gel retardation assays as described in Materials and Methods using the concentrations of protein indicated at the top of the gels. The thin and thick arrows indicate the complexes with high and low mobility, respectively.

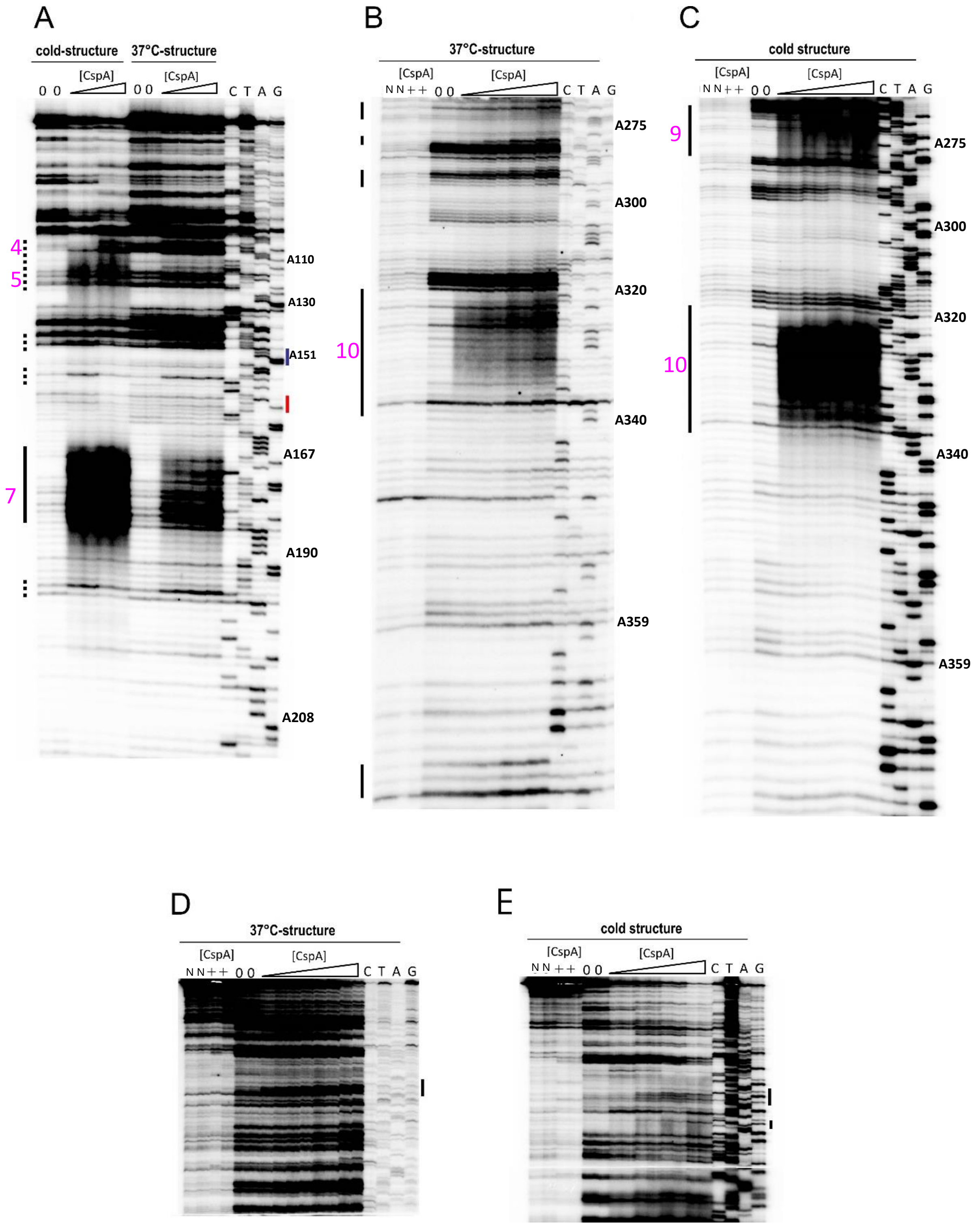

FIGURE S5. Localization of CspA binding sites on *cspA* mRNA by UV-induced cross-linking at 15°C. The experiments were performed in the absence (lanes 0) or in the presence of 28 and 57 µM of CspA (panel A) or 28, 57, 115, 170 µM of CspA (panels B, C, D, and E) with *cspA* mRNA folded in the conformations indicated at the top of the gels. Lanes N: mRNA alone, no cross-links; lanes +: mRNA+170 µM of CspA, no cross-links. Increasing concentrations are indicated with a triangle. All conditions are in duplicate. Lanes C, T, A, and G correspond to sequencing reactions. Primer extension analysis was performed using primer csp2 (panel A), which annealed to the coding region and primer csp3 (panels B, C), which annealed to the 3′ UTR. Panels D and E show the upper parts of the gels displayed in panels B and C. Cross-linked bases are highlighted by black bars on the left side of the gels. These CspA binding sites are numbered (in pink) according to Fig. 3. The blue and red bars on the right side of panel A indicate the Shine-Dalgarno sequence and the AUG initiation triplet, respectively.

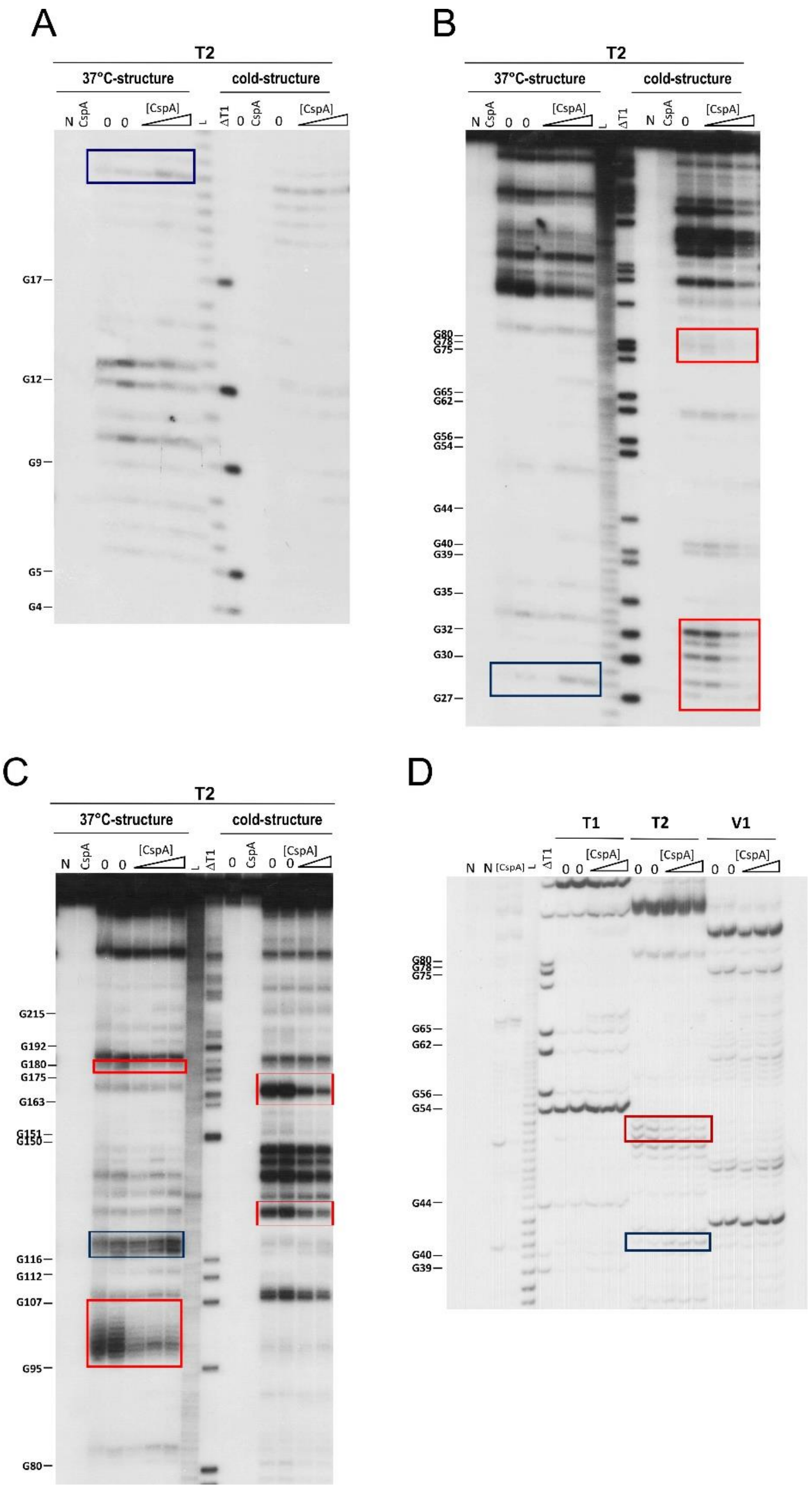

FIGURE S6. Footprinting experiments using RNases. Short (A and B) and long (C) electrophoretic migration of the fragments generated by RNase T2 (T2) digestion of 5′-end [^{32}P]-labelled *cspA* mRNA folded in the conformations indicated at the top of the autoradiographies; (A) bottom and (B) top of the gel. (D) Short electrophoretic migration of the fragments generated by RNases T1, T2, and V1 digestion of 5′-end [^{32}P]-labelled *cspA* mRNA folded in the 37°C-conformation. The experiment was carried out at 15°C in the absence (lanes 0) or in the presence of 80, 102, 160 µM of CspA (increasing concentrations are indicated with a triangle). Lanes N: RNA alone; lanes CspA: RNA+CspA, no RNases; lanes ΔT: RNase T1 cleavages under denaturing conditions; lanes L: alkaline ladder. The red and blue boxes indicate the positions protected or exposed by CspA, respectively.

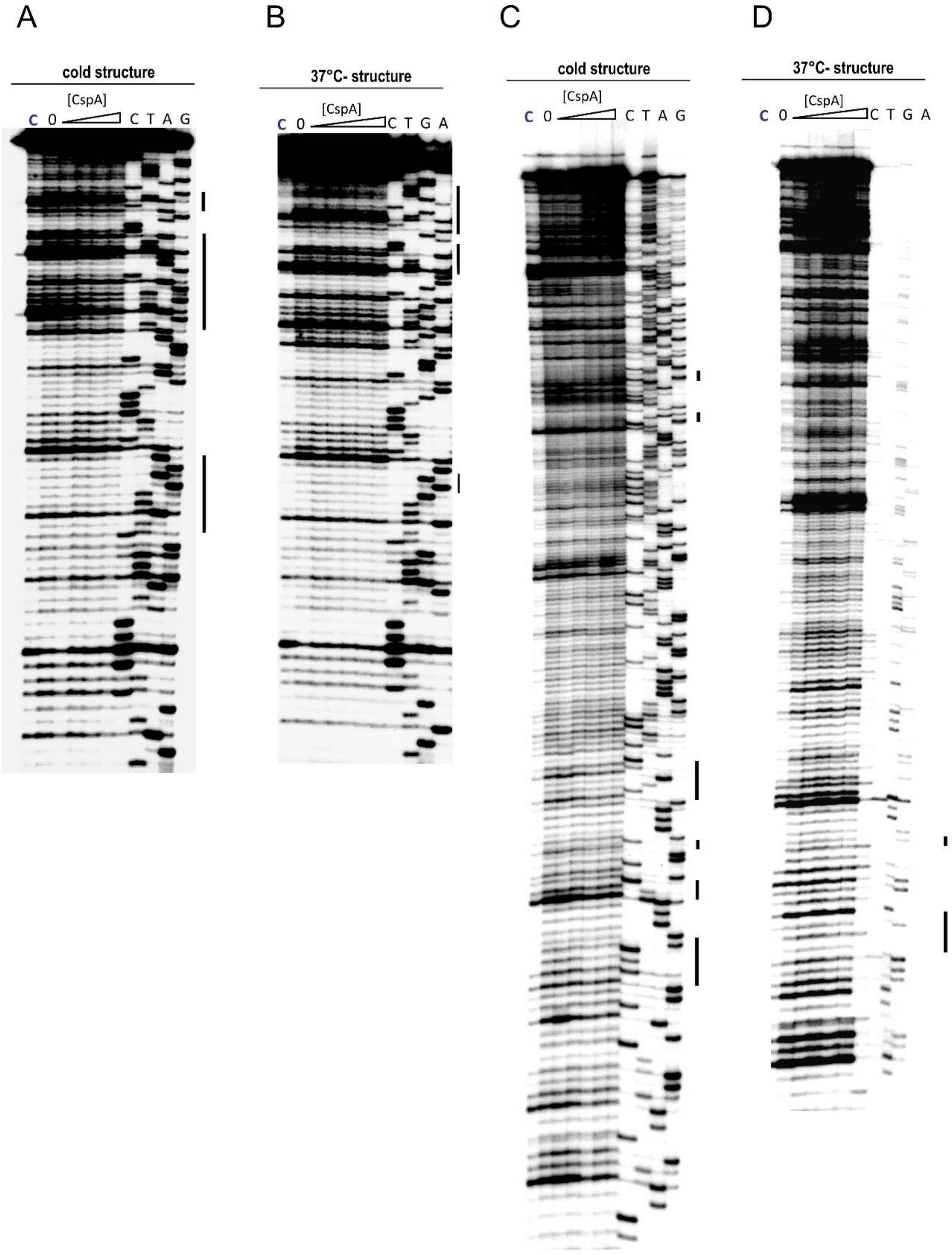

FIGURE S7. Effect of CspA on the *in situ* accessibility of *cspA* RNA to hydroxyl radical cleavage. Primer extension analysis of the cleavage sites generated by hydroxyl radicals on *cspA* mRNA folded in the conformations indicated at the top of the gels, in the presence of increasing concentrations of CspA (0, 15, 30, 60, 120 μM; increasing concentrations are indicated with a triangle). Lanes C: uncleaved mRNA. Lanes C, T, A, and G correspond to sequencing reactions. Primer extension analysis was performed using primer csp1 (panels A and B), which annealed to the 5′ UTR, and primer csp3 (panels C and D), which annealed to the 3′ UTR. Regions whose accessibility to the cleavage is affected by CspA are highlighted by black bars on the right side of the gels.

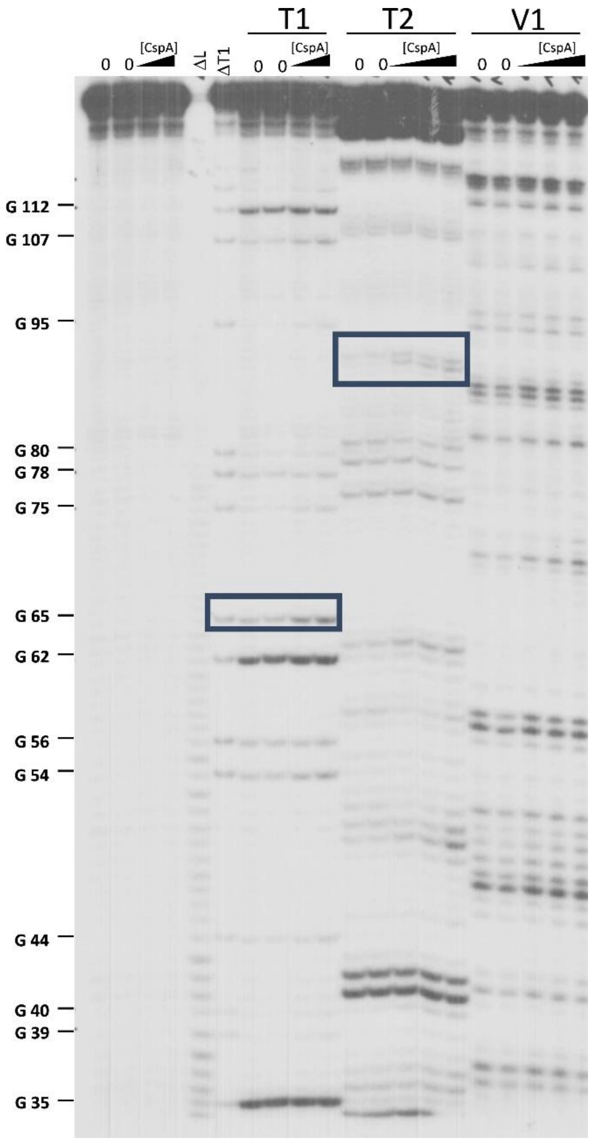

FIGURE S8. Long electrophoretic migration of the fragments generated by RNase T1 (T1), RNase T2 (T2) or RNase V1 (V1) digestion of 5′-end [^{32}P]-labelled 137*cspA* RNA. The experiment was carried out as indicated in Fig. 4.

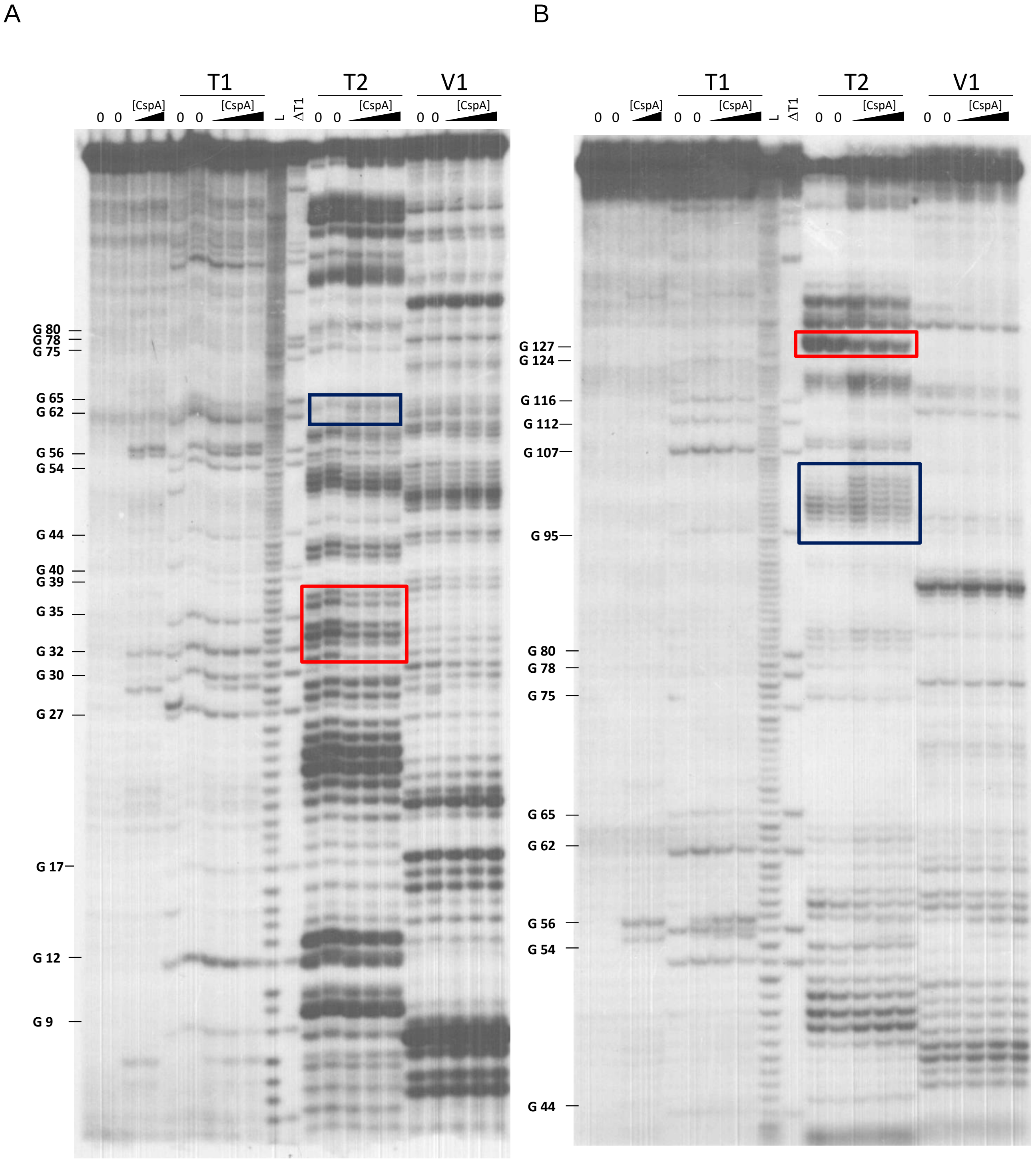

FIGURE S9. Short (A) and long (B) electrophoretic migration of the fragments generated by RNases T1, T2, and V1 digestion of 5′-end [^{32}P]-labelled 187*cspA* mRNA. The experiment was carried out at 37°C in the absence (lanes 0) or in the presence of 80, 102, 160 µM of CspA (increasing concentrations are indicated with a triangle). Lanes ΔT: RNase T1 cleavages under denaturing conditions; lanes L: alkaline ladder. The red and blue boxes indicate the positions protected or exposed by CspA, respectively.

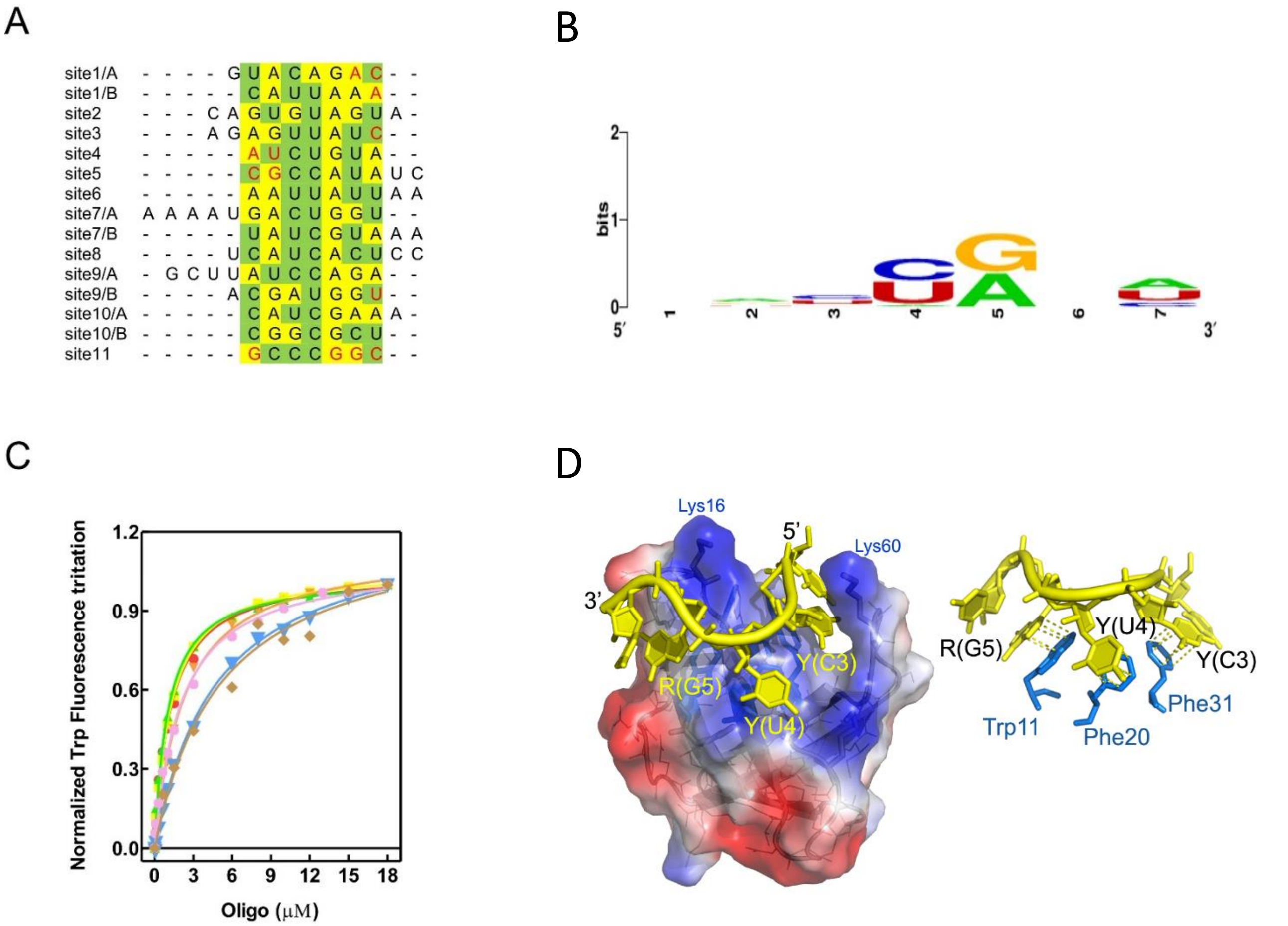

FIGURE S10. Characterization of CspA-RNA interaction. (A) Manual multiple alignment of the sites cross-linked/footprinted by CspA on *cspA* mRNA. Since CspB, the CspA-homologue of *Bacillus subtilis*, recognizes a sequence of 6-7 nts (Lopez and Makhatadze, 2000; Sachs et al., 2012), we hypothesized that the sites in which the cross-links/footprints were particularly extended (> 12 nts) could be the results of the binding of two adjacent CspA molecules. For this reason, we divided these extended sites (sites 1, 7 and 9) into two sub-sequences of comparable length, named A and B, which were used to build the alignment. The yellow and green highlighting indicates purines and pyrimidine, respectively. The bases in red are adjacent to those footprinted/cross-linked by CspA. (B) Logo representation of the CspA binding preference derived from the alignment shown in panel A and generated with WebLogo (Crooks et al., 2004). (C) Quenching of the CspA Trp fuorescence induced by the binding of oligo1 (5′-AACUGGUA-3′, red), which contains the most frequent bases found in the conserved positions of the Logo, or by the following RNA sequences: oligo2 (5′-AAAUGGUA-3′, orange); oligo3 (5′-AACAGGUA-3′, yellow); oligo4 (5′-AACUAGUA-3′, green); oligo5 (5′-AACUGAUA-3′, blue); oligo6 (5′-AACUGGAA-3′, pink) and oligo 7 (5′-AAAAAAAAA-3′, brown). Experiments were performed at 20°C in the presence of 1 µM of CspA and the indicated concentrations of oligos. Further details are given in Materials and Methods. (D) Model of CspA-oligo1RNA interaction obtained using *B. subtilis* CspB-rC7 structure (pdb file 3pf4; Sachs et al., 2012). On the left, CspA protein is visualized with its surface charge distribution (blue positive residues, red negative residues) and oligo1 RNA (yellow) as cartoon representation. The RNA binds in a cleft made by 4 Lys residues: Lys16, Lys10 (not labeled), Lys28 (not labeled) and Lys60. On the right, detailed view slightly rotated compared to the left structure, showing how the Y(C3), Y(U4), R(G5) motif of oligo1 makes stacking interactions with residues Phe31, Phe20 and Trp11, respectively.

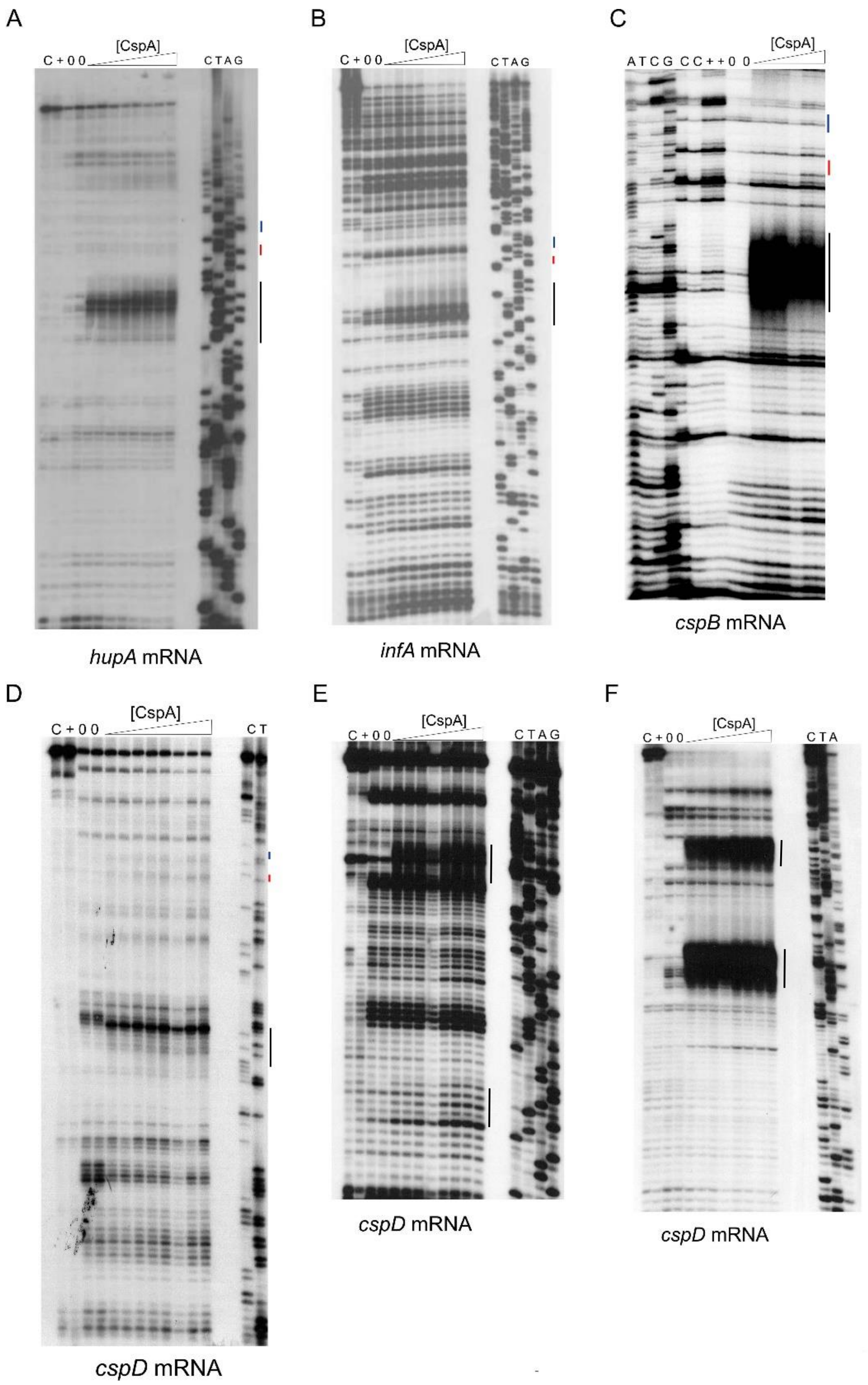

FIGURE S11. Localization of CspA binding sites on *P1infA*, *cspB* and *hupA* and *cspD* mRNAs by UV-induced crosslinking at 15°C. The experiment was performed in the presence of 0, 28, 57, and 120 µM of CspA. Lanes C: mRNA alone, no cross-links; lanes +: mRNA+120 µM of CspA, no cross-links. Increasing concentrations are indicated with a triangle. Lanes C, T, A, and G correspond to sequencing reactions. Primer extension analysis was performed: using primers (A) *hupA* (5′-CTCTGGAGTCCACTCTGGCTG-3′), (B) *P1infA* (5′-GTTCCGCGTAGAGTTAGAAAACG-3′), and (C) *cspB* (5′-GGTAGTAAAGATGTGTTTGTG-3′), which annealed 75, 62, and 70 nts downstream from the initiation codon of the corresponding mRNAs, respectively. Primer extension analysis on *cspD* mRNA was performed using primers: (D) cspD3 (5′-CAGATGGATGGTTACAGAACGC-3′), which annealed in the middle of the coding region, (E) cspD4 (5′-GGAAAAGGGTACTGTTAAGTG-3′) which annealed immediately downstream from the initiation codon, and (F) primer cspD2 (5′-GTCTCATTGTGTACATCCTAAAG-3′), which annealed to the 3′ UTR.
